# Supplementary material for: Protocol of generation of tolerogenic dendritic cells affects their transcriptional and metabolic profiles leading to specific tolerogenic functions
Source: Mol Ther Methods Clin Dev. 2025 Oct 6;33(4):101605. doi: 10.1016/j.omtm.2025.101605 (PMC12589953; doi:10.1016/j.omtm.2025.101605)
Supplement: Document S1. Figures S1 and S2 and Tables S1–S3 [file mmc1.pdf]

OMTM, Volume *33*

Supplemental information

Protocol of generation of tolerogenic dendritic cells affects their transcriptional and metabolic profiles leading to specific tolerogenic functions

Maaike Suuring, Giada Amodio, Mathieu Rouel, Axel Raux, Thomas Delhaye, Anne-Lise Royer, Chloé Cloteau, Denisia Lavercan, David Rondeau, Elise Chiffoleau, Francesca Santoni de Sio, Mikaël Croyal, Silvia Gregori, and Aurélie Moreau

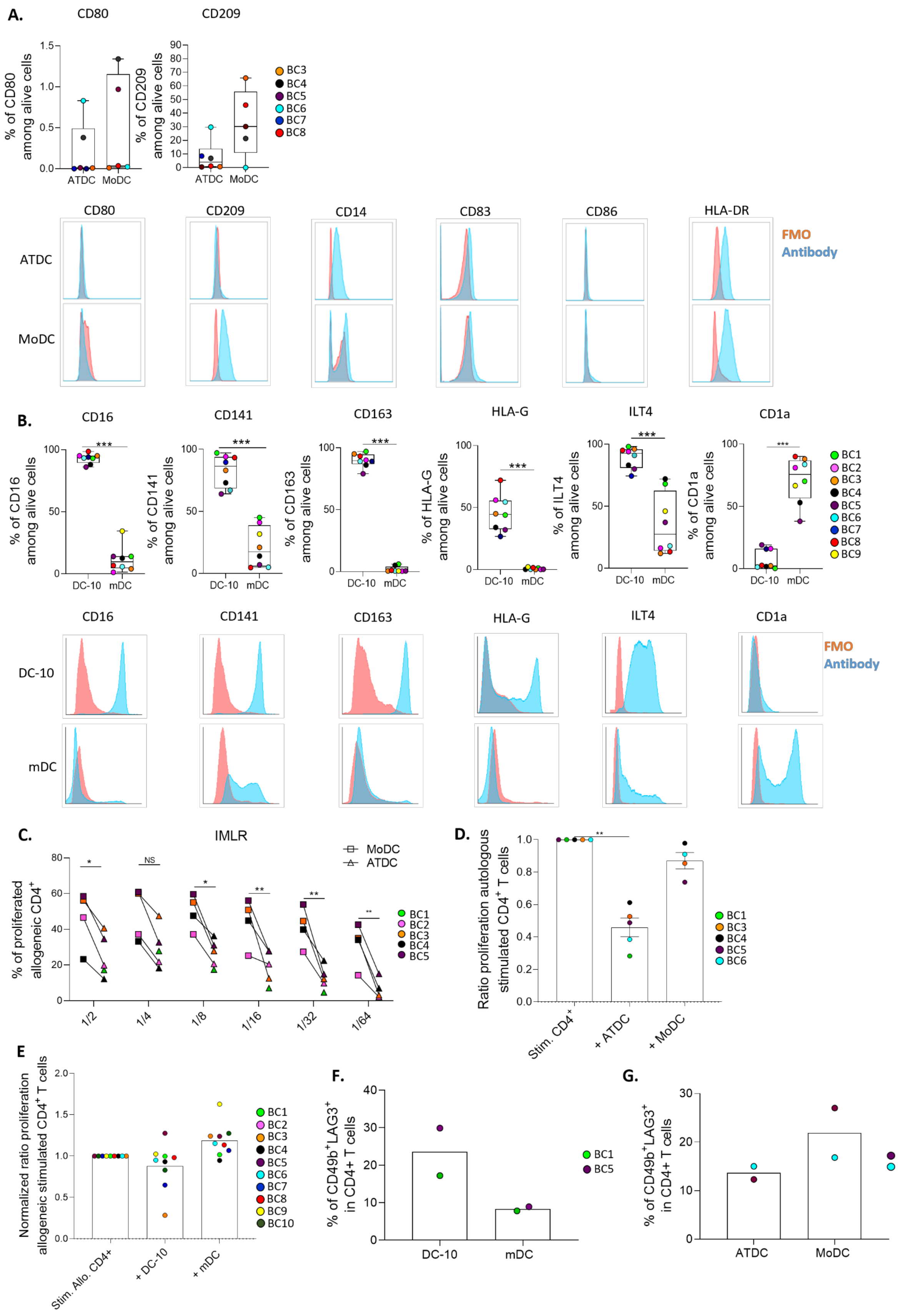
A.
CD80
CD209
% of CD80 among alive cells
% of CD209 among alive cells
ATDC
MoDC
BC3
BC4
BC5
BC6
BC7
BC8
CD80
CD209
CD14
CD83
CD86
HLA-DR
ATDC
MoDC
FMO
Antibody
B.
CD16
CD141
CD163
HLA-G
ILT4
CD1a
% of CD16 among alive cells
% of CD141 among alive cells
% of CD163 among alive cells
% of HLA-G among alive cells
% of ILT4 among alive cells
% of CD1a among alive cells
DC-10
mDC
***
BC1
BC2
BC3
BC4
BC5
BC6
BC7
BC8
BC9
CD16
CD141
CD163
HLA-G
ILT4
CD1a
DC-10
mDC
FMO
Antibody
C.
IMLR
MoDC
ATDC
% of proliferated allogeneic CD4+
*
NS
**
1/2
1/4
1/8
1/16
1/32
1/64
BC1
BC2
BC3
BC4
BC5
D.
Ratio proliferation autologous stimulated CD4+ T cells
Stim. CD4+
+ ATDC
+ MoDC
BC1
BC3
BC4
BC5
BC6
E
Normalized ratio proliferation allogeneic stimulated CD4+ T cells
Stim. Allo. CD4+
+ DC-10
+ mDC
BC1
BC2
BC3
BC4
BC5
BC6
BC7
BC8
BC9
BC10
F.
% of CD49b+LAG3+ in CD4+ T cells
DC-10
mDC
BC1
BC5
G.
% of CD49b+LAG3+ in CD4+ T cells
ATDC
MoDC
BC5
BC6

Figure S1 – ATDC and DC-10 display distinct phenotype and tolerogenic functions *(related to Figure 2)*

(A) Frequency (%) of cells expressing the indicated markers among alive cells in ATDC and MoDC ($n \geq 5$ donors). Histograms of the markers expressed by ATDC and MoDC (blue) and their respective FMO controls (orange). **(B)** Bar charts of the frequency (%) of cells expressing the indicated markers among alive cells in DC-10 and mDC ($n = 8$ donors). **(C)** DCs were cultured with allogeneic CPD labeled $CD4^+$ T cells at the indicated T:DC ratios. At day 5, T cell proliferation was assessed by CPD dilution ($n \geq 6$ donors). **(D and E)** CPD labeled $CD4^+$ T cells were cultured with allogeneic mature DCs at 1:10 T:DC ratio in the presence or absence of ATDC and MoDC (panel D) or DC-10 and mDC (panel E). At day 5, T cell proliferation was assessed by CPD dilution ($n = 5$ donors). **(F and G)** $CD4^+$ T cells were cultured with allogeneic DCs, either DC-10 or mDC (panel F), or ATDC or MoDC (panel G), at 1:10 ratio for 10 days. At the end of the culture the frequency of Tr1 cells was analyzed by the co-expression of LAG-3 and CD49b by flow cytometry ($n = 2$ donors). p values were calculated by Mann-Whitney T-test (A - B), two-way ANOVA (C) and Kruskal-Wallis test (D)*$p < 0.05$, **$p < 0.01$,***$p < 0.001$.

A.

DC-10 VS ATDC

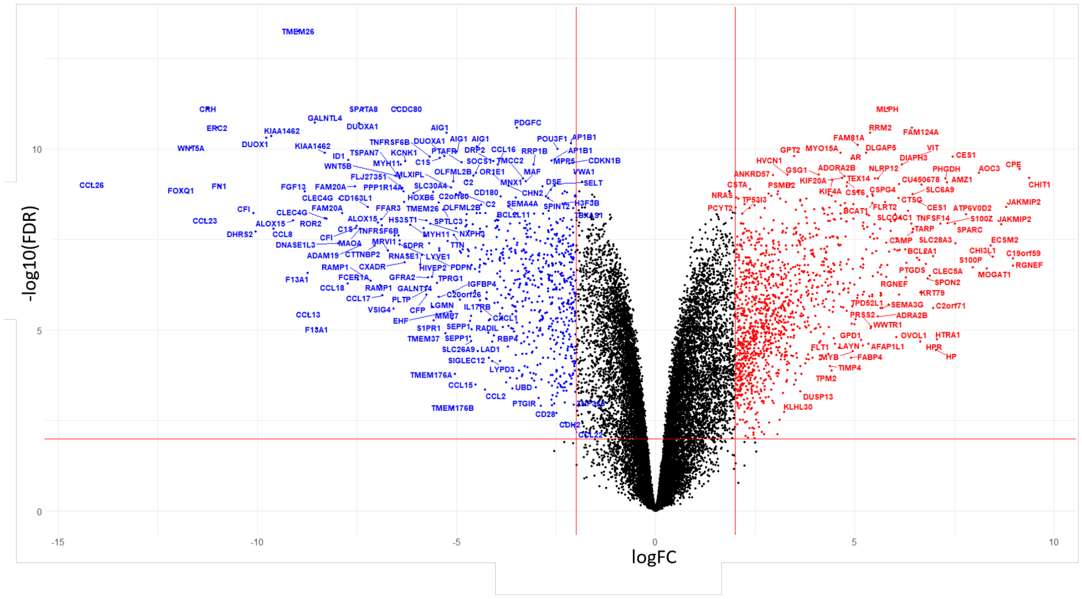

B.

MoDC VS ATDC

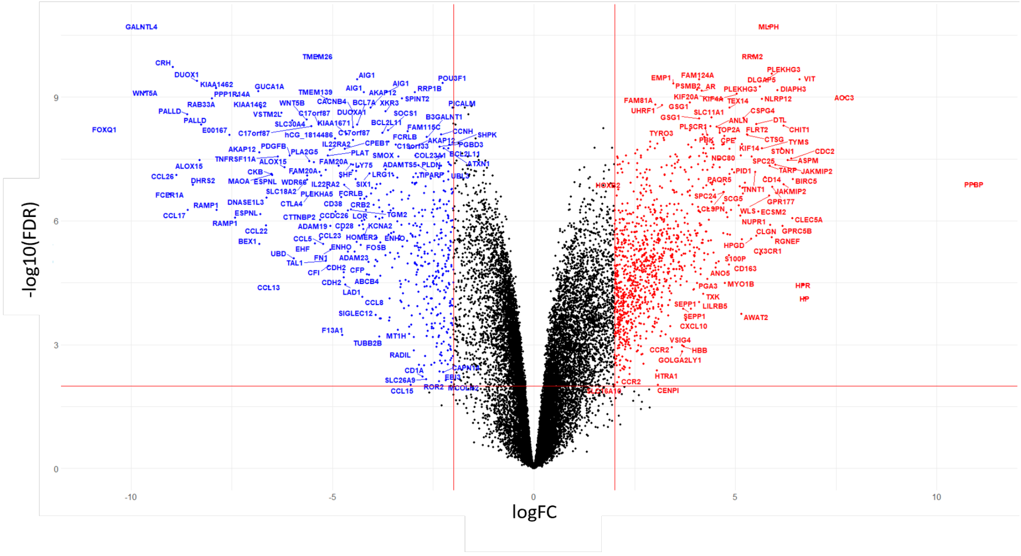

MoDC VS DC-10

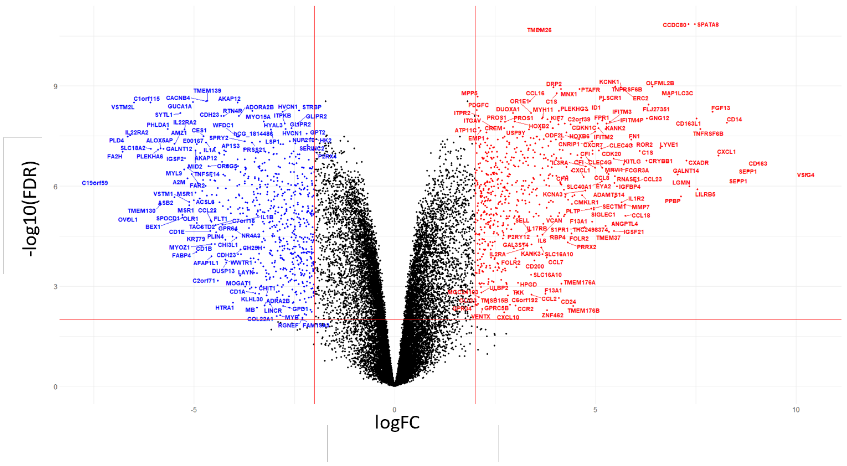

Figure S2 –Volcano plots associated to the transcriptomic analyses *(related to Figure 5)*

(A) Volcano plot illustrates the genes differentially expressed between ATDC and DC-10 ($n \geq 6$ donors). Genes upregulated in DC-10 are depicted on the left (blue). **(B)** Volcano plots illustrate the genes differentially expressed between either ATDC (up) or DC-10 (down) and MoDC ($n \geq 6$ donors). Genes upregulated in MoDC are depicted on the left (blue).

Table S1 – Multiple Reaction monitoring parameters used for the quantification of amino acids by mass spectrometry.

Amino acid	Parent ion (m/z	Fragment ion (m/z)	Cone (V)	Collision (eV)
Aspartate	246.1	144	30	20
^{15}N-aspartate (IS)	247.1	145	30	20
Glutamate	260.1	83.9	30	20
^{15}N-Glutamate (IS)	261.1	84.9	30	20
Alanine	146.1	56.9	30	15
^{13}C-Alanine (IS)	147.1	56.9	30	15
Arginine	231.1	69.9	30	20
^{13}C-Arginine (IS)	232.1	69.9	30	20
Asparagine	189.1	73.9	30	20
$^{15}N_2$-Asparagine (IS)	191.1	74.9	30	20
Cysteine	178.1	75.9	30	15
d_3-Cysteine (IS)	181.1	78.9	30	15
Glutamine	203.1	83.9	30	20
^{15}N-Glutamine (IS)	204.1	84.9	30	20
Glycine	132.1	56.9	30	15
^{13}C-Glycine (IS)	133.1	56.9	30	15
Histidine	212.2	109.9	30	20
$^{15}N_3$-Histidine (IS)	215.2	112.9	30	20
Isoleucine / Leucine	188.2	85.9	30	15
^{15}N-Leucine (IS)	189.2	86.9	30	15
Lysine	203.1	83.9	30	20
$^{13}C_6$-Lysine (IS)	209.1	88.9	30	15
Methionine	206.2	103.9	30	15
^{13}C-d_3-methionine (IS)	210.2	107.9	30	15
Phenylalanine	222.2	120.2	30	20
d_5-Phenylalanine (IS)	227.2	125.2	30	20
Proline	172.1	69.9	30	20
^{13}C-Proline (IS)	173.1	70.9	30	20

Serine	162.1	59.9	30	15
^{15}N-Serine (IS)	163.1	60.9	30	15
Threonine	176.1	73.9	30	15
^{15}N-Threonine (IS)	177.1	74.9	30	15
Tryptophane	261.2	132	30	20
d_5-Tryptophane (IS)	266.2	137	30	20
Tyrosine	238.2	136	30	20
^{15}N-Tyrosine (IS)	239.2	137	30	20
Valine	174.1	71.9	30	20
^{13}C-Valine (IS)	175.1	71.9	30	20

IS, internal standard.

Table S2 – Multiple Reaction monitoring parameters used for the quantification of sphingolipids by mass spectrometry.

Sphingolipids	Parent ion (m/z	Fragment ion (m/z)	Cone (V)	Collision (eV)
Cer (d18:1/16:0)	538.5	264.3	28	26
Cer (d18:1/18:0)	566.5	264.3	30	26
Cer (d18:1/20:0)	594.3	264.3	28	26
Cer (d18:1/22:0)	622.6	264.3	30	30
Cer (d18:1/24:0)	650.6	264.3	34	26
SM (d18:1/16:0)	703.6	184.1	58	26
SM (d18:1/18:0)	731.5	184.1	58	32
SM (d18:1/20:0)	759.7	184.1	46	26
SM (d18:1/22:0)	787.7	184.1	40	30
SM (d18:1/24:0)	815.7	184.1	36	30

IS, internal standard.

Table S3 – Multiple Reaction monitoring parameters used for the quantification of acylcarnitines by mass spectrometry.

**Acylcarnitines**	**Parent ion (m/z**	**Fragment ion (m/z)**	**Cone (V)**	**Collision (eV)**
Ethyl-carnitine (C2)	260.4	85.1	30	21
Propanoyl-carnitine (C3)	274.4	85.1	30	21
Butyryl-carnitine (C4)	288.4	85.1	30	21
Lauroyl-carnitine (C12)	400.5	85.1	30	24

IS, internal standard.
