# Supplementary material for: Protocol of generation of tolerogenic dendritic cells affects their transcriptional and metabolic profiles leading to specific tolerogenic functions
Source: Mol Ther Methods Clin Dev. 2025 Oct 6;33(4):101605. doi: 10.1016/j.omtm.2025.101605 (PMC12589953; doi:10.1016/j.omtm.2025.101605)
Supplement: Document S2. Article plus supplemental information [file mmc2.pdf]

Molecular Therapy
Methods & Clinical Development
Original Article

Protocol of generation of tolerogenic dendritic cells affects their transcriptional and metabolic profiles leading to specific tolerogenic functions

Maaike Suuring,[1,2,8] Giada Amodio,[3,8] Mathieu Rouel,[1,2] Axel Raux,[4] Thomas Delhaye,[5] Anne-Lise Royer,[4] Chloé Cloteau,[6] Denisia Lavercan,[7] David Rondeau,[5] Elise Chiffoleau,[1,2] Francesca Santoni de Sio,[3] Mikaël Croyal,[6,7] Silvia Gregori,[3,9] and Aurélie Moreau[1,2,9]

[1]Nantes Université, INSERM, Center for Research in Transplantation and Translational Immunology, UMR 1064, ITUN, 44000 Nantes, France; [2]LabEx IGO, Nantes Université, 44000 Nantes, France; [3]Mechanisms of Peripheral Tolerance Unit, San Raffaele Telethon Institute for Gene Therapy (SR-TIGET), IRCCS San Raffaele Scientific Institute, 20132 Milan, Italy; [4]MELISA Core Facility, Oniris, LABERCA INRAE, 44307 Nantes, France; [5]University Rennes, CNRS, Institut d'Electronique et des Technologies du numéRique – UMR 6164, SM[2] Platform, 35000 Rennes, France; [6]Nantes Université, CNRS, INSERM, L'institut du Thorax, 44000 Nantes, France; [7]Nantes Université, CHU Nantes, Inserm, CNRS, SFR Santé, Inserm UMS 016, CNRS UMS 3556, 44000 Nantes, France

Cell therapy is a promising approach for inducing tolerance and thus reducing the use of immunosuppressive drugs in several immune-mediated conditions. Tolerogenic dendritic cells (tolDCs) regulate antigen-specific tolerance and control the immune response, making them an interesting candidate for cell therapy. Over the last years, different human tolDCs have been generated *ex vivo*, each with specific phenotypic and functional characteristics. This study aims to highlight the impact of the manufacturing process on tolDC function. For that, two well-known tolDCs, autologous tolerogenic dendritic cells (ATDCs) and interleukin (IL)-10-induced dendritic cells (DC-10s), were compared based on their phenotype, metabolic secretion, transcriptomic profile, and their modulatory function. Our findings indicate that both ATDCs and DC-10s effectively regulate T cell responses; however, while DC-10s accomplish this through immune regulation, ATDCs rely on metabolic adaptations. Understanding these specific regulatory mechanisms is critically important in order to select the best disease to target using these tolDCs as cell-based therapy.

INTRODUCTION

The improvement of cell manufacturing protocols and the accomplishment of the first clinical trials using cell-based therapy in recent years have paved the way for the clinical application of tolerogenic cells for the treatment of immunological diseases. Several approaches of cell-based therapy have been developed, including mesenchymal stem/stromal cells,[1–8] regulatory T cells (Tregs),[9–12] peripheral blood mononuclear cells (PBMCs),[13,14] and antigen-presenting cells (APCs).[15–22] Among the different mechanisms underlying tolerance induction by the indicated cells, APCs exert multiple immunosuppressive effects by inhibiting effector T cell activation/proliferation and by promoting Tregs. This regulation by APCs is mediated by contact-dependent and independent mechanisms. Among APCs, tolerogenic dendritic cells (tolDCs) have been recognized as a promising tool for cell-based immunotherapy due to their ability to promote antigen-specific tolerance and regulate immune responses.[23,24] TolDCs are generated *ex vivo* from peripheral blood monocytes and are characterized by low expression of costimulatory molecules, such as CD80 and CD86, along with upregulation of inhibitory and modulatory receptors (i.e., PD-L1, ILT3/4). TolDCs secrete low levels of pro-inflammatory cytokines and high levels of anti-inflammatory cytokines (e.g., interleukin-10 [IL-10] and transforming growth factor β [TGF-β]).[25] TolDCs present antigens to T cells through major histocompatibility complex (MHC)-T cell receptor interaction, but the low co-stimulation associated with inhibitory signals leads to T cell hypo-responsiveness and clonal anergy.[26] Importantly, the overall potency of tolDCs is linked to their ability to inhibit effector T cell responses and to induce Tregs.[23,27] Although this study is performed on dendritic cells (DCs) generated *ex vivo* for possible application in cell-based therapy, results might also be important for better understanding the physiological process leading to tolDC differentiation *in vivo*. Indeed, the impact of the microenvironment, including nutrients (such as glucose or lactate), cytokines (such as IL-10 and IL-4), and growth factors (such as granulocyte-macrophage colony stimulating factor [GM-CSF] or macrophage colony stimulating factor) on the differentiation of tolDCs is not well studied but is a major concern.

Several human tolDCs have been generated using growth factors, cytokines, genetic modifications, and pharmacological agents in order to enhance their regulatory properties.[28–38] Although these different

Received 21 January 2025; accepted 1 October 2025;
https://doi.org/10.1016/j.omtm.2025.101605.

[8]These authors contributed equally
[9]Senior author
Correspondence: Aurélie Moreau, Nantes Université, INSERM, Center for Research in Transplantation and Translational Immunology, UMR 1064, ITUN, 44000 Nantes, France.
E-mail: aurelie.moreau@univ-nantes.fr

Published by Elsevier Inc. on behalf of The American Society of Gene and Cell Therapy.
This is an open access article under the CC BY license (http://creativecommons.org/licenses/by/4.0/).

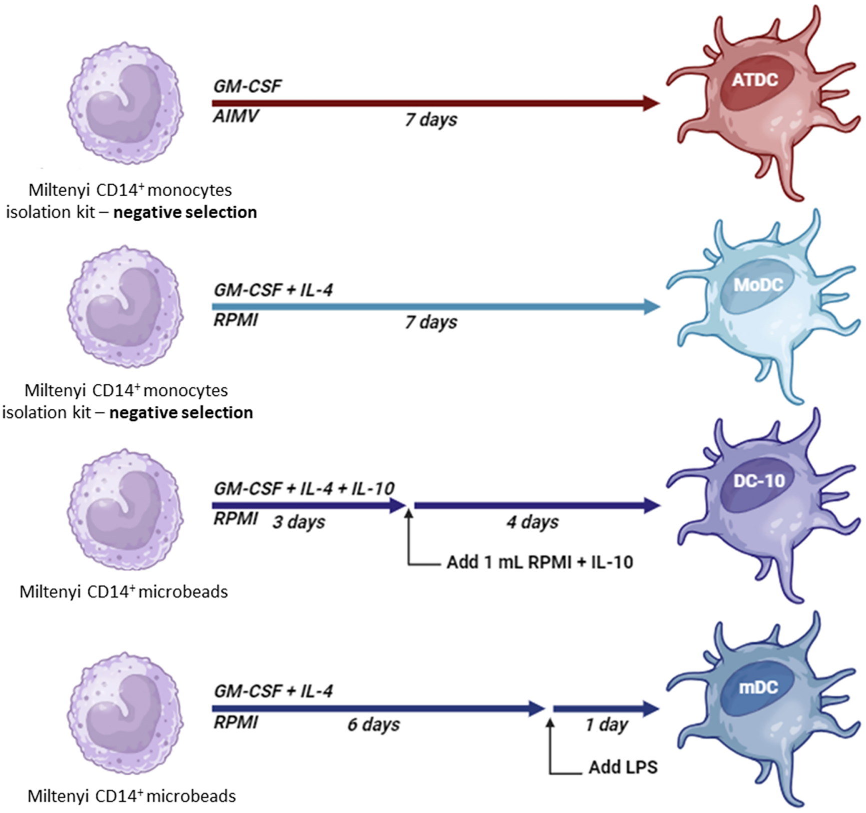

Figure 1. Protocols used to generate ATDCs, DC-10s, MoDCs, and mDCs

ATDCs and MoDCs were generated from untouched monocytes, whereas DC-10s and mDCs were generated from positively selected CD14$^+$ monocytes. ATDCs were differentiated with GM-CSF in AIMV medium. The other DCs were differentiated in complete RPMI/FCS medium with GM-CSF and IL-4 for MoDCs; GM-CSF, IL-4, and IL-10 for DC10s; and GM-CSF, IL-4, and LPS for mDCs. All cells were cultured for 7 days. More details can be found in the materials and methods section. Created with Biorender.

tolDCs exhibit distinct features and properties, we aimed to investigate whether they also share some similarities. TolDC characterizations were done separately in distinct laboratories. Thus, we aimed to explore whether reported differences can be related to their generation or if inherent mechanisms of regulation occur due to external factors (like operator or location-dependent variability). In this study, we investigated two *ex vivo*-generated human tolDCs: autologous tolerogenic dendritic cells (ATDCs) and IL-10-induced dendritic cells (DC-10s), initially developed by the team of Moreau (France) and Gregori (Italy), respectively. ATDCs are generated with a low dose of GM-CSF in the absence of IL-4 and exhibit low expression of CD80, CD83, CD86, and human leukocyte antigen (HLA)-DR. ATDCs inhibit T cell proliferation, expand FOXP3$^+$ Tregs, and are resistant to maturation stimuli.[39–42] A first phase 1/2 clinical trial was performed with ATDCs as a treatment to minimize immunosuppressive drugs in kidney transplantation. This trial was shown to be safe and feasible with promising results.[15,41] DC-10s are generated with GM-CSF, IL-4, and IL-10; these cells express CD86 and HLA-DR, along with inhibitory molecules, like ILT3, ILT4, and HLA-G. DC-10s induce hypo-responsive allogeneic T cells and promote type 1 regulatory T (Tr1) cells.[38,43]

In the present work, ATDCs and DC-10s were generated from the same donors. Our objectives, after the validation of ATDC and DC-10 phenotype, their stimulatory activity, and their regulation of T cell responses as previously published,[27,38] were to compare their ability to secrete or consume metabolites and their transcriptome. ATDCs display an immature phenotype, while as expected, DC-10s show a semi-mature phenotype, and both tolDCs modulate T cell responses. However, from the metabolic and transcriptomic profiles, ATDCs and DC-10s shared some regulatory mechanisms but also distinct features.

RESULTS

ATDCs and DC-10s are distinct tolDCs sharing the ability to modulate primary T cell responses

To properly compare ATDCs and DC-10s, we generated tolDCs and control DCs from peripheral blood monocytes of the same donors (n = 10) in the same laboratory. As previously described,[27] ATDCs were differentiated from untouched monocytes with a low dose of GM-CSF (100 U/mL) in AIMV medium, and their internal control, monocyte-derived DCs (MoDCs), were differentiated following a classical protocol with GM-CSF and IL-4 in complete RPMI/fetal calf serum (FCS) medium (Figure 1). As previously described,[38] DC-10s were generated from positively selected CD14$^+$ monocytes cultured with GM-CSF, IL-4, and IL-10 in complete RPMI/FCS medium, and their internal control, mature DCs (mDCs), were differentiated with GM-CSF, IL-4, and lipopolysaccharide (LPS) (Figure 1). On day 7, DCs were collected and analyzed for their phenotype and ability to modulate T cell responses. To validate ATDC and DC-10 functional activity, we used their internal controls (MoDCs and mDCs, respectively) as previously described.[27,38] These two control cells differed in the step of maturation (LPS stimulation was present only in mDCs).

Upon collection, the yield recovery and viability of ATDCs were high and, as expected, DC-10s showed low yield recovery and viability, due to the use of IL-10 during the differentiation, which is known to exert a pro-apoptotic effect[44] (Figures 2A and 2B). In line with previous studies,[27,38] ATDCs displayed an immature phenotype (CD14$^+$CD83$^-$CD86$^{-/low}$HLA-DR$^{-/low}$), whereas DC-10s displayed a semi-mature phenotype (CD14$^+$CD83^{low}CD86^{int}HLA-DR$^+$), suggesting a high capacity of antigen presentation by the latter cells (Figure 2C; Table 1). TolDC phenotypes were validated using

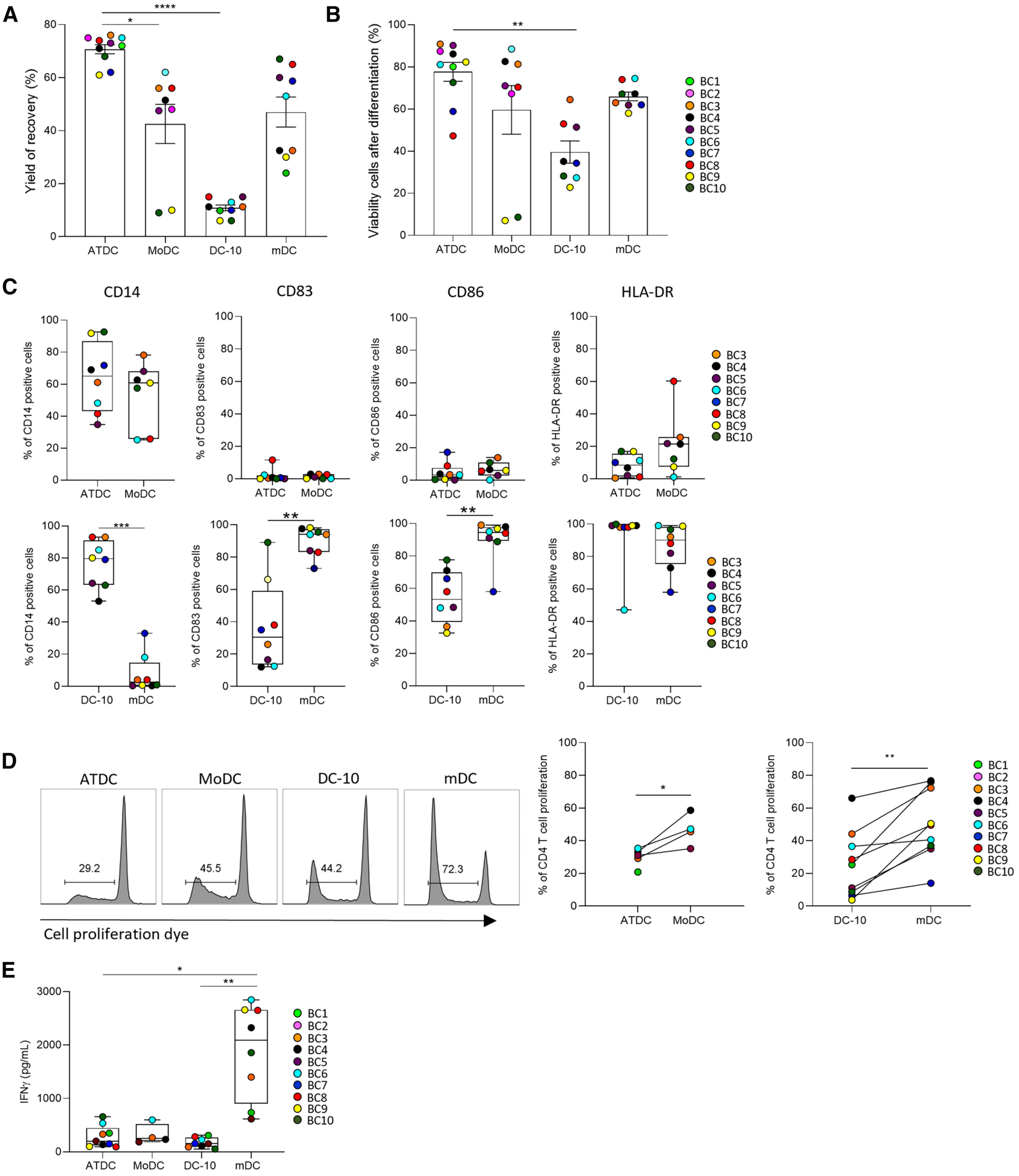

Figure 2. ATDCs and DC-10s display different phenotypes but both modulate T cell responses

ATDCs, DC-10s, and their relative controls were differentiated from monocytes from the same donors according to the protocol described in the materials and methods. At day 7, DCs were collected and their phenotype and functions were analyzed. (A) Yield of recovery and (B) viability of cells after differentiation were measured in the different DC populations ($n \geq 9$ donors, Buffy Coat 1 to Buffy Coat 10). (C) Frequencies of cells (%) expressing the indicated markers among alive cells were quantified in ATDCs, MoDCs, DC-10s, and mDCs by flow cytometry ($n \geq 5$ donors). (D) DCs were cultured with allogeneic $CD4^+$ T cells at 1 ATDC:8 T cells ratio or 1 DC-10:10 T cells ratio, and T cell proliferation was assessed after 5 days by proliferation dye dilution ($n \geq 5$ donors). (E) IFNγ concentration was measured by ELISA in the supernatants of DC/T cell MLR assay described in (D) ($n \geq 5$ donors). p values were calculated by Kruskal-Wallis test (A, B, and E), Mann-Whitney t test (C), and Wilcoxon t test (D). $^*p < 0.05$, $^{**}p < 0.01$, $^{***}p < 0.001$.

additional specific markers: ATDCs were $CD209^{-}CD80^{-/low}$, while DC-10s were $CD16^{+}CD141^{+}CD163^{+}ILT4^{+}HLA\text{-}G^{+}CD1a^{-/low}$ (Figures S1A and S1B). As expected, MoDCs displayed an immature phenotype and expressed HLA-DR at higher levels compared to ATDCs (Figure 2C). It is worth noting that MoDCs from some donors (donors 4 to 6) express low levels of CD209, suggesting an

Table 1. Average relative fluorescence intensity of CD14, CD83, CD86, and HLA-DR surface markers expressed by the DC populations from 9 donors

	CD14		CD83		CD86		HLA-DR	
	RFI	SD	RFI	SD	RFI	SD	RFI	SD
ATDCs	12, 5	±11, 7	5, 7	±4, 2	1, 9	±4, 1	4, 3	±1, 8
MoDCs	5, 3	±3, 8	3, 8	±2, 4	4, 5	±9, 5	4, 7	±1, 8
DC-10s	29, 6	±14, 0	1, 8	±0, 8	1, 8	±0, 2	9, 4	±3, 4
mDCs	0, 4	±0, 6	20, 1	±14, 1	63, 9	±65, 5	9, 7	±5, 0

RFI, relative fluorescence intensity; SD, standard deviation.

incomplete differentiation (Figure S1A). Conversely, mDCs showed a shift of maturation with a high expression of CD83, CD86, and HLA-DR markers and loss of CD14 (Figure 2C). We then investigated the immune-modulatory ability of tolDCs using a mixed lymphocyte reaction (MLR) assay, confirming that both ATDCs and DC-10s induced low proliferative responses of allogeneic $CD4^+$ T cells compared to their respective controls, in agreement with their tolerogenic profiles (Figures 2D and S1C). According to their hypo-stimulatory ability, both ATDCs and DC-10s did not promote interferon gamma (IFNγ) production by allogeneic $CD4^+$ T cells (Figure 2E). As previously shown, ATDCs suppressed the proliferation of $CD4^+$ T cells induced by allogeneic mDCs (Figure S1D),[27] while DC-10s did not (Figure S1E). Furthermore, in line with previous studies,[43] we validated the ability of DC-10s to promote the induction of $CD49b^+LAG3^+$ Tr1 cells (Figure S1F), while ATDCs did not (Figure S1G). Altogether, these analyses confirmed previously published data on ATDCs[27] and DC-10s[38,43] and demonstrated that, although ATDCs and DC-10s display distinct phenotypes and mechanisms of modulation of T cell responses, they share the ability to promote hypo-responsiveness of allogeneic $CD4^+$ T cells.

ATDCs and DC-10s are characterized by common and specific metabolites

To define and compare the metabolic profile of the two subsets of tolDC, at the end of their differentiation, ATDCs, DC-10s, and their relative controls, MoDCs and mDCs, were collected and cultured for an additional week in RPMI/FCS medium in the absence of cytokines or growth factors. At the end of this second culture, supernatants were collected to analyze the metabolites secreted or consumed by these DCs. RPMI/FCS medium alone was used as a control of the basal level for each metabolite. An untargeted metabolomic analysis has been first performed using traditional mass spectrometry-based approaches. A partial least squares-discriminant analysis (PLS-DA) was plotted from all detected signals to illustrate the distinct profiles of ATDC and DC-10 metabolomes (Figure 3A). Metabolomic fingerprints of ATDCs and DC-10s did not overlap and were primarily separated from the second component (Dim. 2 on Figure 3A). MoDCs and mDCs exhibited a closely related metabolomic profile. Although RPMI/FCS condition appeared closer distributed to mDCs on this 2D PLS-DA, these two populations were well segregated by component 3 in a 3D PLS-DA plot (Figure 3B), indicating a specific metabolomic profile generated by mDCs. Taking advantage of the human metabolome database, a classification of the features based on their molecular family revealed a high percentage of lipids, including acylglycerols, glycerophospholipids, and sphingolipids, as well as a high amount of amino acids and acylcarnitines (Figure 3C). To formally confirm the observations of the untargeted analysis, the concentrations of amino acids, acylcarnitines, and sphingolipids were quantified in the same samples by targeted mass spectrometry-based assays. Regarding the non-essential and essential amino acids presented in Figures 4A and 4B, respectively, we observed that glutamine was less consumed in both ATDCs and DC-10s compared to their internal controls, suggesting that other sources of nutrients are used by tolDCs (Figure 4A). Furthermore, less alanine was measured in tolDC supernatants compared to other DC supernatants, suggesting a lower production of this amino acid. Notably, alanine is a glucogenic amino acid as it is synthetized from pyruvate by alanine aminotransferase. Lastly, a higher secretion of glycine was detected in tolDCs compared to control DCs (Figure 4A). There were also some differences between ATDCs and DC-10s. Indeed, serine and cysteine were significantly more consumed by ATDCs compared to DC-10s (Figure 4A). The consumption of these two amino acids could suggest a different regulation of folate or glutathione pathways in ATDCs. Originally, most of the essential amino acids (lysine, valine, threonine, phenylalanine, leucine, histidine, and methionine), as well as tyrosine, were secreted in higher amount by DC-10s compared to their secretion by ATDCs (Figures 4A and 4B). Generally, a higher secretion of amino acids was measured in DC-10 supernatant compared to other DCs, and even in some cases higher than RPMI/FCS medium, suggesting potential storage of amino acids by DC-10s during their differentiation process. No major differences were observed in the measurements of sphingolipids, either ceramides or sphingomyelins, among the different DC subsets (Figures 4C and 4D). About the carnitines, a similar higher consumption of propanoyl-carnitine was observed in tolDCs compared to mDCs, whereas only ATDCs significantly consumed butyryl-carnitine. Interestingly, a strong secretion of ethyl-carnitine was observed only in DC-10s (Figure 4E). Lastly, a significantly higher lactate concentration was measured in ATDC and DC-10 supernatants compared to their respective controls (Figure 4F). Altogether, even if these targeted and quantitative analyses show that ATDCs and DC-10s displayed distinct secretomes, a metabolic fingerprint associated with glycolysis (low consumption of

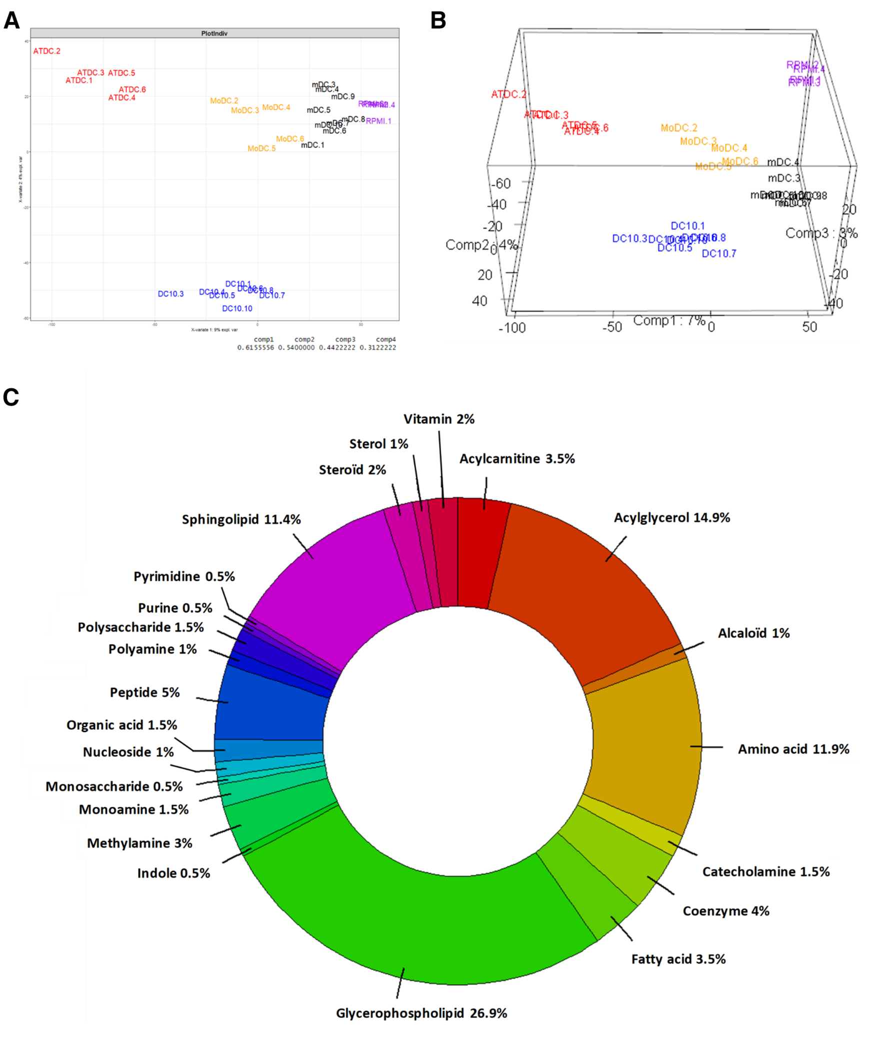

Figure 3. Metabolomic profiles differ between DC types

After differentiation, ATDCs, MoDCs, DC-10s, and mDCs were harvested and cultured in RPMI/FCS medium. Supernatants were collected after 7 days and analyzed by mass spectrometry using untargeted or targeted methods to define their metabolic profiles. RPMI/FCS medium (RPMI) was used as control of basal level of each metabolite. (A) The plot depicts the partial least squares-discriminant analysis (PLS-DA) of metabolomic data (unsupervised analysis) ($n \geq 6$ donors). (B) The plot depicts the 3D PLS-DA of unsupervised metabolomic analysis ($n \geq 6$ donors). (C) Pie chart illustrates the metabolic families detected in the unsupervised metabolomic analysis.

glutamine, low production of alanine, and strong production of lactate) appears in both tolDCs. These metabolomic analyses open the way for further investigations, including metabolic flux analysis with stable isotopes, to decipher the role of specific metabolites in the stability of ATDCs and DC-10s and the involvement of other metabolites in the tolerogenic functions of these cells.

ATDCs and DC-10s have a distinct transcriptional profile

We next compared the transcriptomic profiles of ATDCs and DC-10s. MoDCs were used as control DCs since they were differentiated in the absence of pro-tolerogenic agents and were not activated with LPS (as ATDCs and DC-10s). The multidimensional scaling (MDS) plot demonstrated that ATDCs, DC-10s, and MoDCs were well separated based on their gene expression (Figure 5A). In agreement with the MDS, 2,591 differentially expressed genes between ATDCs and DC-10s were identified on the volcano plot (false discovery rate <0.01 et log2FC > 2 ou < −2) (Figure S2A). Kyoto Encyclopedia of Genes and Genomes (KEGG) pathway analysis performed on all genes showed an enrichment of three pathways linked to cell replication (i.e., cell cycle, motor protein, and cytoskeleton pathways) in ATDCs compared to DC-10s (Figure 5B). Specifically, this analysis revealed the upregulation of genes involved in various aspects of cell cycle (*PKL1*, *CCNB1*, and *CDKN2B*) and cytoskeletal organization (integrins [*ITGA6* and *ITGA7*], *MYL2*, *MYL9*, *tropomyosin* [*TPM1* and *TPM2*], *TUBB2A*) (Figure 5C). The six other enriched pathways observed in ATDCs were associated with cellular metabolism, including oxidative phosphorylation (OXPHOS), amino acids, lipids, nucleotides, and carbons (Figure 5B). Regarding lipids, upregulated genes were associated with fatty acid metabolism (*ACOX2*) and, more specifically, with the uptake (*CD36*), transport (*CD36*, *CPT1A*), and catabolism of fatty acids (*ACADSB*) that can fuel the tricarboxylic acid (TCA) cycle in ATDCs (Figure 5C). The observation of the overexpression of some enzymes (*ACCS1/2* and *PDHA1*) also suggests that acetate or pyruvate might represent substrates for the TCA cycle. In ATDCs, we also noticed a significant number of upregulated genes directly linked to oxidative phosphorylation, such as electron transport chain (ETC) complex I (*NDUFS1*, *NUDFS2*, *NUDFS6*, and *NDUFS7*), ETC complex III (*UQCRC1* and *CYC1*), ETC complex IV (*COX7A2L*, *COX7B*, and *COX11*), and ETC complex V (*ATP6V1E2*, *ATP6-VOD2*, *ATP5G1*, *ATP5J2*, and *ATP6AP1*) (Figure 5C). The pathways enriched in DC-10s compared to ATDCs were all linked to immune responses (i.e., allograft rejection, antigen processing and presentation, cytokine-cytokine receptor interaction, and signaling pathways) (Figure 5B). As expected, DC-10s overexpressed IL-10 and its pathway, including *IL10RA* and *STAT3*, but also *EBI3* and *TGFBR2*, which mediates TGF-β signaling that is essential for maintaining immune tolerance. In agreement with the phenotypic analysis, MHC class II molecules were overexpressed in DC-10s (*HLA-DQB1*, *HLA-DPB1*, *HLA-DPA1*, *HLA-DMA*, and *HLADRB5*), as well as chemokines involved in immune cell recruitment and

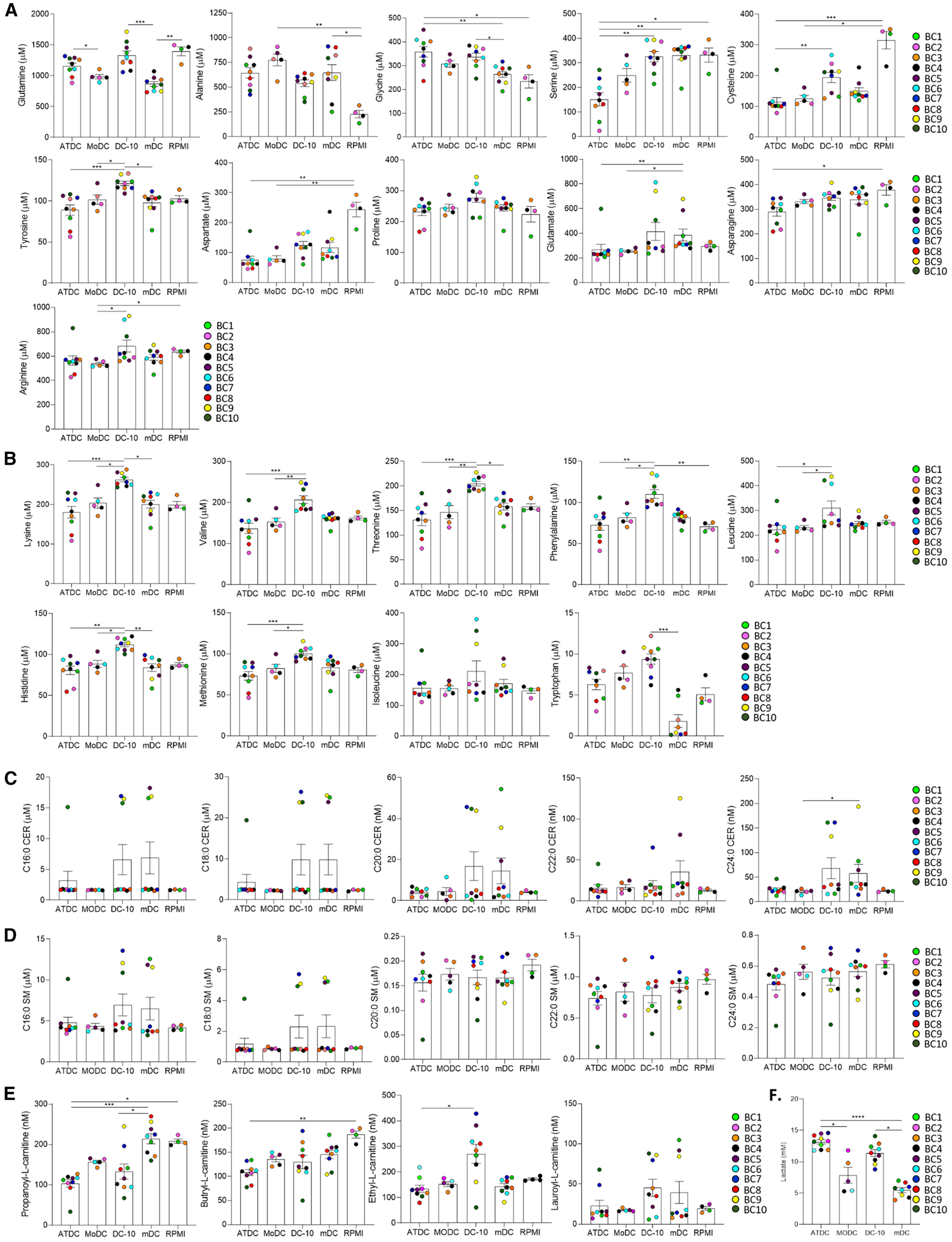

(legend on next page)

migration (*CCL19*, *CCL5*, *CCL8*, *CCL4*, *CCL26*, *CXCR4*, *CXCR3*, and *CXCR7*) and interleukin receptors regulating T cell response and regulation (*IL2RA*, *IL15RA*, and *IL4R*). Moreover, DC-10s also expressed *CSF1R*, which promotes DC survival and differentiation (Figure 5C). Comparison of either ATDCs or DC-10s versus MoDCs revealed 25 common up-regulated genes and 13 common down-regulated genes in tolDCs (Figures 5D and S2B). Up-regulated genes included some chemokines (*PPBP* alias *CXCR7*, *CCR2*, *CXCL1*, *CXCL3*, and *CXCL10*), anti-inflammatory molecules or genes associated to a reduction of pro-inflammatory signaling (*IL1R2*, *TNFSF10* alias *TRAIL*, *SERPING1*, and *SERPINA1*), genes associated to pathogen-associated molecular pattern (PAMP) recognition pathways (*CD14*, *TLR7*, and *NOD2*), and the regulation of coagulation system (*SERPING1*, *PROS1*, and *PROCR*). Other genes were linked to cholesterol efflux and lipid metabolism (*ABCA1*) or management of oxidative stress (*CYBB*) (Figure 5D, left). The commonly down-regulated genes were mainly related to cell adhesion (integrins *ITGB7* and *ITGB8*), cadherin (*CDH2*), and ion transport (*ATP1B2*, *MCOLN2*, and *P2RX5*). Interestingly, *CD28* and *CTLA4*, two genes coding for the ligands of B7 family but with opposite actions on T cells, were down-regulated in both ATDCs and DC-10s in comparison with MoDCs. Lastly, one gene associated with lipid degradation (*PLCB1*) and another associated with serotonin receptor (*HTR2B*) were also down-regulated (Figure 5D, right). Altogether, these data demonstrate the heterogeneity between ATDCs and DC-10s, which possibly reflects their distinct tolerogenic properties. Indeed, ATDCs are more prone to modulate the metabolic microenvironment, which drives their tolerogenic properties, as previously demonstrated with lactate,[27] whereas DC-10s are more effective in modulating the immune response via several mechanisms.

DISCUSSION

Here, we aimed at highlighting shared and/or unique features underlying the tolerogenic properties of ATDCs and DC-10s. In this respect, cells were differentiated from the same donors and compared in term of phenotype, function, metabolic, and transcriptomic profiles. Results confirmed that ATDCs and DC-10s are phenotypically different, induce allogeneic T cell hypo-responsiveness *in vitro*, and display specific modulatory functions. ATDCs and DC-10s share the ability to produce lactate and glycine and consume a lower amount of glutamine compared to control DCs. They are also characterized by specific metabolic profiles, with ATDCs being more prone to consume serine and cysteine and DC-10 to secrete essential amino acids, tyrosine, and ethylcarnitine. ATDCs and DC-10s also have a distinct transcriptional profile, with ATDCs seeming more prone to modulate their cellular metabolism, whereas DC-10s appear more effective in modulating the immune response.

One major difference between ATDCs and DC-10s is cell survival. Upon harvesting, our data indicated that ATDCs were more viable and therefore showed a higher recovery yield compared to DC-10s. While the low DC-10 cell recovery is due to the pro-apoptotic effect of IL-10 on human monocytes, as already described in the literature,[44–46] the upregulation of genes associated with the cell cycle and some genes of motor proteins detected only in the ATDC transcriptome support the overall high ATDC cell recovery after differentiation. Further experiments are required to study the proliferative ability of ATDCs. Furthermore, the pathway associated with the cytoskeleton was also upregulated in ATDCs. The cytoskeletal genes modulate the migratory ability of DCs to lymphoid tissues and interaction with T cells. Even though the genes are not directly regulating immunoregulatory pathways, they may be involved in important cellular processes for the tolerogenic properties of ATDCs.

DC-10s, in comparison with ATDCs, express higher levels of co-stimulatory molecules and HLA class II molecules, making them more prone to induce antigen-specific tolerance. This phenotypic difference is supported by the up-regulation of antigen presentation molecules in DC-10s compared to ATDCs. Indeed, the antigen processing and presentation pathway is enriched in DC-10 transcriptomic profile compared to the ATDC one. The higher antigen-presenting capacity of DC-10s is also linked to their unique ability to promote Tr1 cells,[47] which are known to be antigen specific.[48] ATDCs display a low expression of HLA molecules and costimulatory or activation markers, consistent with their transcriptomic profile. Indeed, all pathways associated with effector immune response are clearly down-regulated in ATDCs compared to DC-10s. These data confirm previous reports describing ATDCs as immature DCs, whereas DC-10s display a semi-mature phenotype.[27,38] It is important to highlight that ATDCs are generated in the absence of IL-4, which could affect their transcriptome and metabolome and also potentially favor possible macrophage differentiation. Indeed, we previously reported that ATDCs shared markers with DCs and tolDCs (such as CD1c, CD141, ILT2, and ILT3) but also with macrophages (such as CD64 and CX3CR1), as well as a shared transcriptomic profile.[27] However, our previous results demonstrated that their tolerogenic properties, such as regulation of T cell proliferation and activation, Treg generation, as well as production of anti-inflammatory cytokines and also resistance to maturation stimuli induced by several Toll-like receptor ligands, clearly relate them to tolDCs and not to macrophages.[27] In this paper, ATDCs are phenotypically similar to MoDCs (except for CD209) and appear to be closer to them in terms of the immune response pathways than DC-10s and MoDCs.

ATDCs and DC-10s share some up-regulated genes related to cell migration (CCR7) or PAMP recognition, highlighting their capacity to migrate *in vivo* to secondary lymph nodes and to sense the

Figure 4. ATDCs and DC-10s exhibit common and specific metabolites

(A–E) Non-essential amino acids (A), essential amino acids (B), ceramides (C), sphingomyelins (D), and acylcarnitines (E) were quantified by targeted mass spectrometry analyses in supernatants of ATDCs, MoDCs, DC-10s, and mDCs. (F) Lactate concentration (mM) was measured in supernatants of ATDCs, MoDCs, DC-10s, and mDCs using lactate meter system ($n \geq 6$ donors). Values were calculated by Kruskal-Wallis test (C–F). *$p < 0.05$, **$p < 0.01$, ***$p < 0.001$.

A

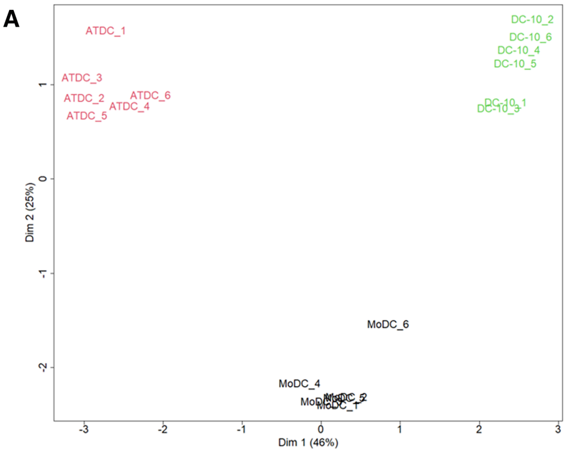

B

KEGG pathways enriched in ATDC compared to DC-10

Gene sets	NES	p-adjust
Cell cycle	1,90	0,0001
PPAR signaling pathway	1,78	0,0095
Pyrimidine metabolism	1,71	0,0208
Fatty acid metabolism	1,70	0,0258
Oxidative phosphorylation	1,68	0,0064
Biosynthesis of amino acids	1,62	0,0258
Motor proteins	1,61	0,0130
Carbon metabolism	1,57	0,0316
Cytoskeleton in muscle cells	1,48	0,0444

KEGG pathways enriched in DC-10 compared to ATDC

Gene sets	NES	p-adjust
Allograft rejection	2,02	0,0073
Antigen processing and presentation	1,93	0,0030
Cytokine-cytokine receptor interaction	1,86	0,0001
Complement and coagulation cascades	1,85	0,0091
Chemokine signaling pathway	1,81	0,0006
Th1 and Th2 cell differentiation	1,80	0,0091
IL-17 signaling pathway	1,62	0,0444
Th17 cell differentiation	1,59	0,0495

C

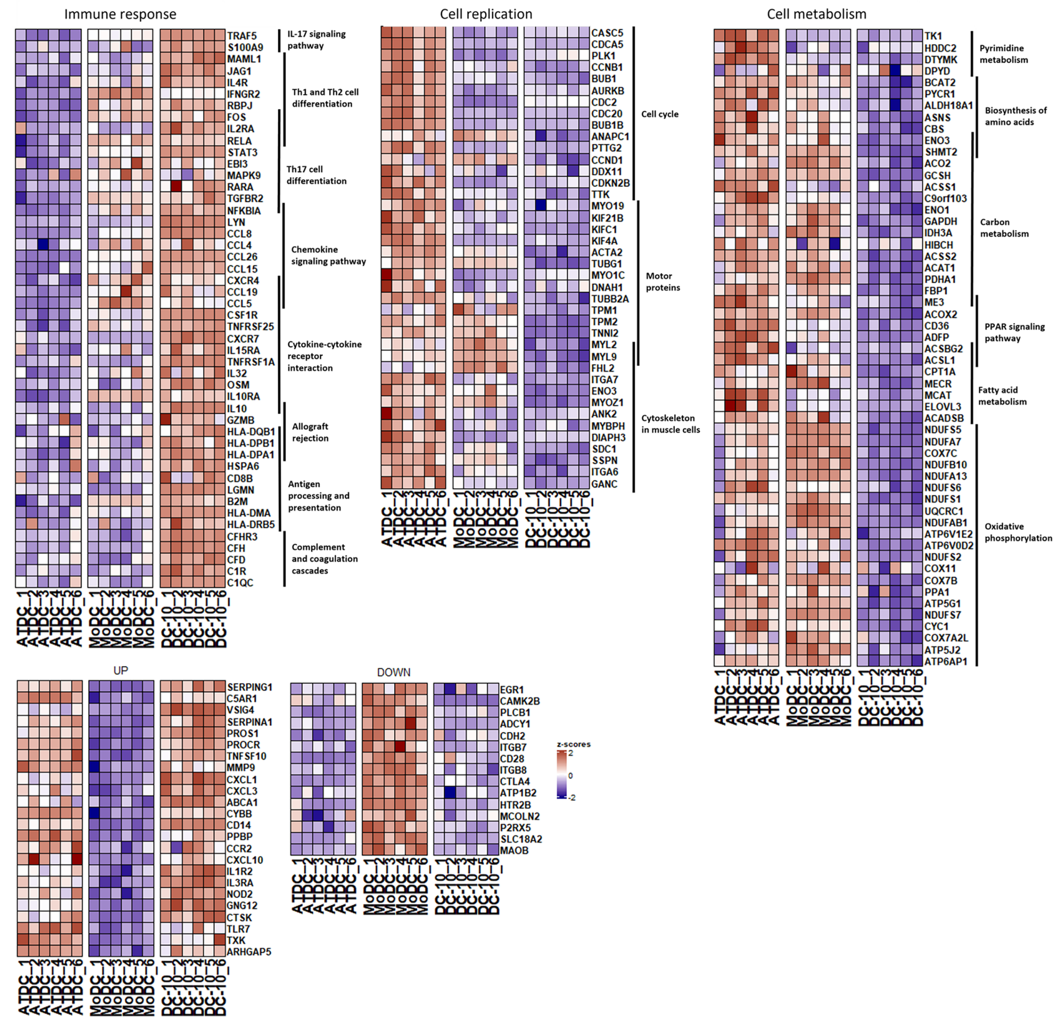

environment. Moreover, both ATDCs and DC-10s induced allogeneic T cell hypo-responsiveness and the ability to inhibit IFNγ production by T cells. The last major difference between ATDCs and DC-10s in the transcriptomic analysis concerns their cellular metabolism, which is enriched in ATDCs. This result is consistent with our previously published data demonstrating that ATDCs display a strong glycolytic and oxidative metabolism.[27] It can be postulated that the higher cellular metabolism in ATDCs reflects their higher cell viability/cell recovery compared to DC-10s. Accordingly, these metabolic pathways are down-regulated in DC-10s.

We previously published that ATDCs regulated $CD4^+$ T cells thanks to their specific cellular metabolism. More precisely, we discovered that lactate was responsible for the suppression of $CD4^+$ T cell proliferation. ATDCs also promote Treg expansion by unknown secreted metabolite(s) but independently of lactate.[27] Metabolomic fingerprints of ATDCs and DC-10s highlighted that these two tolDC populations are distinct. In contrast to genomic and transcriptomic analyses that refer to uncontroversial databases, the identification of metabolites is challenging because there are several ways to detect a small molecule by mass spectrometry, and each molecule has its own signal, which depends on the instrument. Measuring consumed/secreted amino acids highlighted that DC-10 secreted a high amount of amino acids (even more than the medium for some). These data suggest an accumulation of amino acids, including essential ones, in DC-10s. One possible explanation is the ability of DC-10s to perform autophagy or efferocytosis of apoptotic monocytes. Further experiments will be required to investigate this hypothesis. The possible recycling of nutrients by DC-10s is in line with the low metabolic activity of these cells observed in transcriptomic analysis. Such metabolic regulation mechanisms can also explain the low recovery yield of DC-10s. In contrast, ATDCs secrete low amounts of amino acids, reflecting their high cellular metabolism. Further investigations are required to decipher whether the low amount of secreted serine (and cysteine) detected in ATDCs can be associated with an overproduction of glutathione, which protects the cell from oxidative stress. ATDCs and DC-10s also produced some common metabolites, including lactate and glycine that were previously described for their immunomodulatory properties.[27,49–51] It is worth noting that lactate has been described to alter gene expression in myeloid cells by epigenetic modifications.[52] Such epigenetic changes can contribute to long-term maintenance of tolerance.[53] Lactate secretion is one of the characteristics associated with other subtypes of *in vitro* differentiated tolDCs, such as those generated in the presence of vitamin D3 alone or in combination with dexamethasone. Indeed, VitD3DC and VitD3/Dexa-DCs showed a stable OXPHOS program associated with an overall higher glycolytic capacity (reviewed in Morali et al.[54]). In VitD3DC, CD115 signaling promotes metabolic reprogramming, leading to enhanced glycolysis and glucose consumption and production of lactate.[55]

This study has limitations. Since we compared ATDCs and DC-10s generated from the same donors, all the cells were differentiated in the same laboratory (Gregori lab). ATDCs and MoDCs were thus generated in a new environment, which can explain possible changes in their phenotype. Indeed, we noticed some differences in the phenotype and function of MoDCs used in this study compared to our previous report.[27] More precisely, in this study, MoDCs displayed a lower expression of co-stimulatory molecules, an intermediate expression of CD209, and produced a higher amount of lactate (±7 mM) compared to previous observations. This difference could affect the outcome of the metabolic comparison. A second limitation concerns the metabolomic analysis, which was performed in an untargeted manner. This approach allowed for the generation of an unbiased overview of the metabolome, but the identification of the metabolites was a difficult process. Targeted analyses of some amino acids, acylcarnitines, and sphingolipids were performed based on major outcomes of the untargeted approaches. Still, additional targeted analyses (i.e., organic acids, nucleotides, and biogenic amines) are required to fully compare the metabolic profiles of ATDCs and DC-10s.

In conclusion, this study confirms that ATDCs and DC-10s are efficient in modulating T cell responses but highlights the different mechanisms of action of these cells. Data suggest that ATDCs regulate responses and mediate tolerance via metabolic adaptations or by the secretion of small immunomodulators, whereas DC-10s control T cell responses and promote tolerance in an antigen-specific manner and by the secretion of cytokines/proteins. Interestingly, both tolDCs showed a proper metabolic regulation including the secretion of several shared metabolites and displayed a different transcriptomic profile with few genes commonly expressed. Further studies are warranted to fully explore and compare the regulatory capacity of ATDCs and DC-10s and to understand more in depth the precise mechanisms of the metabolic adaptation used by these tolDCs.

MATERIALS AND METHODS

Generation of ATDCs, MoDCs, DC-10s, and mDCs

Dendritic cells were generated from human PBMCs, from healthy volunteer donors (men/women) in accordance with local committee approval (TIGET09) and the Declaration of Helsinki. PBMCs were isolated by density gradient centrifugation over Lympholyte-H. Monocytes were isolated by magnetic labeling via positive selection or negative selection following the manufacturer's instructions. ATDCs were differentiated following a 7-day culture of 1×10^6

Figure 5. ATDCs and DC-10s present a distinct transcriptional profile

DNA microarrays were performed on ATDCs, DC-10s, and mDCs generated from monocytes of the same 6 donors. (A) The plot depicts the MDS of these DC populations (*n* = 6 donors). (B) Tables showing the KEGG pathways enriched in ATDCs (in comparison to DC-10s) or in DC-10s (in comparison to ATDCs). (C) Heatmap illustrates the core enriched genes for each pathway identified in KEGG enrichment analysis (*n* = 6 donors). (D) Heatmap illustrates the common genes expressed by ATDCs and DC-10s in comparison to MoDCs (*n* = 6 donors).

monocytes/mL in AIMV medium CTS supplemented with recombinant human GM-CSF at 100 U/mL in a 6-well plate. For the generation of MoDCs, 1×10^6 monocytes/mL were seeded in a 6-well plate in 5 mL of complete medium (RPMI 1640 medium containing 10% FCS, 1% L-glutamine, 1% antibiotics, 1 mM sodium pyruvate, 1 mM HEPES, and 1% non-essential amino acids) supplemented with recombinant human IL-4 (200 U/mL) and recombinant human GM-CSF (100 U/mL) for 7 days. DC-10s were differentiated following a 7-day culture in complete RPMI medium at 1×10^6 monocytes/mL supplemented with recombinant human IL-4 (10 ng/mL), recombinant human GM-CSF (100 ng/mL), and recombinant human IL-10 (10 ng/mL) in a 24-well plate for 7 days. On day 3, 1 mL/well of pre-warmed medium with cytokines, at vaforementioned concentrations, was added. mDCs were obtained following the DC-10 protocol but avoiding the use of IL-10 and matured at day 6 with 1 μg/mL of LPS. All DCs were harvested on day 7 for phenotypical and functional assays.

T cell assays

The function of DCs was tested by means of MLR and suppression assays. For both assays, $CD4^+$ T cells were isolated from PBMCs by negative selection using the human $CD4^+$ T cell Isolation Kit II according to the manufacturer's instructions. $CD4^+$ T cells were labeled with a Proliferation Dye eFluor 670 according to the manufacturer's instructions for 20 min at 37°C. In MLR assay, allogeneic $CD4^+$ T cells were cocultured in a round-bottom 96-well plate with DCs at a ratio of 1:10 for DC-10s and at different ratios with ATDCs. In suppression assay, labeled $CD4^+$ T cells were stimulated with allogeneic mDCs and cocultured with tolDCs at a ratio of 1 T cell:0, 1 allogeneic mDC:1 tolDC. All cell cultures were incubated at 37°C with 5% CO_2. The T cell proliferation of both assays was measured on day 5 by flow cytometry with the following antibodies: anti-CD3 and anti-CD4. Flow cytometry was performed on an FACS Canto II or an FACS LSR and analyzed with FlowJo or FCS Express software.

Tr1 induction protocol

$CD4^+$ T cells were purified from PBMCs by negative selection using the human $CD4^+$ T cell Isolation Kit II according to the manufacturer's instructions. $CD4^+$ T cells were labeled with Cell Proliferation Dye eFluor 670 according to the manufacturer's instructions and stimulated with 1×10^4 allogeneic DC-10s or mDCs (10:1, T:DCs) in X-VIVO 15 medium supplemented with 5% human serum and 100 U/mL penicillin/streptomycin. After 10 days, primed T cells were collected, washed, and analyzed for phenotype by flow cytometry. The phenotype of resulting T cells was analyzed using the following antibodies: anti-CD3, anti-CD4, anti-CD45RA, anti-CD25, anti-HLA-DR, anti-CD49b, and anti-LAG-3. Dead cells were excluded using fixable viability dye eFluor450 or eFluor506. Flow cytometry was performed on an FACS Canto II or an FACS LSR (BD Biosciences) and analyzed with FlowJo or FCS Express software.

Lactate measurements

At the end of their differentiation, ATDCs, DC-10s, MoDCs, and mDCs were collected and cultured for 1 additional week in RPMI/FCS medium in the absence of cytokines or growth factors. Supernatants were then collected, and lactate production was quantified in the supernatants with lactate test strips.

Agilent microarray data

ATDCs, DC-10s, and MoDCs were generated from CliniMACS-sorted $CD14^+$ human monocytes, obtained by leukapheresis from six male healthy donors according to the methods described in "cell generation" section. RNAs were extracted from cultured cells using the RNeasy Plus Mini kit and then labeled and hybridized on Agilent whole-genome oligo Microarrays (8 × 60K) according to the "One-Color Micro-array-Based Gene Expression analysis" protocol. The raw data are deposited in GEO: GSE278825.

All the downstream analyses were managed with the edgeR package. The raw intensity dataset was filtered to remove probes with low intensities, reduce noise, and improve the statistical power of the analysis. After filtering, the data were normalized using the trimmed mean of M component method, and an MDS plot was generated to represent the samples in a lower dimensional space. Dispersion estimates were calculated to accurately account for variability in gene expression levels across samples before fitting the generalized linear model (GLM). This step ensures that the statistical analysis properly considers inherent biological and technical variability, leading to more reliable identification of differentially expressed genes. Gene set enrichment analysis) was performed with the KEGG database using the GLM test results to identify enriched pathways. Similar upregulated and downregulated genes between ATDCs and DC-10s, compared to MoDCs, were identified according to specific threshold and analyzed using the KEGG database for over-representation analysis. This allowed for the identification of common regulated pathways between the two cell types.

Metabolomics analysis

At the end of their differentiation, ATDCs, DC-10s, MoDCs, and mDCs were collected and cultured for 1 additional week in RPMI/FCS medium in the absence of cytokines or growth factors. Supernatants were then collected and frozen at −80° to analyze the metabolites. As a control, complete RPMI medium was incubated for 7 days in the same conditions. A quality control (QC) sample was prepared by pooling 25 μL from each individual sample and subsequently split into several aliquots.

Metabolites were extracted from individual samples and QCs by protein precipitation. Ice-cold methanol (800 μL) was added to defrost samples (200 μL). Following 10 s vortex, the mixture was centrifuged (20,000 × g, 10 min, 4°C), and the clear supernatant was split in two equal fractions of 300 μL. All samples were transferred to liquid chromatography-mass spectrometry (LC-MS) vial to be evaporated to dryness under a gentle nitrogen stream (room temperature). Dried samples were reconstituted either with 5% acetonitrile (fraction 1, 100 μL) or with 75% acetonitrile (fraction 2, 100 μL), each containing 0.1% formic acid. All solvents were purchased from Biosolve. Metabolomic analysis was conducted by liquid chromatography-high-resolution mass spectrometry analyses on a Synapt G2 HRMS Q-TOF

mass spectrometer equipped with an electrospray ionization (ESI) interface operating in both the positive (ESI+) and the negative (ESI−) ionization mode and an Acquity H-Class UPLC device (Waters Corporation). Individual samples were randomized and injected (5 μL) altogether with QC samples, either onto a reversed-phase column (HSS T3, Waters Corporation, 2.1 × 100 mm, 1.7 μm, fraction 1) held at 60°C or an hydrophilic interaction liquid chromatography (HILIC) column (BEH-amide, Waters Corporation, 2.1 × 100 mm, 1.7 μm, fraction 2) held at 45°C. Metabolites from fraction 1 were separated with a linear gradient of mobile phase B (0.1% formic acid in acetonitrile) in mobile phase A (0.1% formic acid in water) at a flow rate of 400 μL/min. Mobile phase B was kept constant at 1% for 1 min, linearly increased from 1% to 95% for 15 min, kept constant for 2 min, returned to the initial condition over 1 min, and kept constant for 5 min before the next injection. Metabolites from fraction 2 were separated with a linear gradient of mobile phase A (10 mmol/L ammonium acetate, 0.1% formic acid) in mobile phase B (98% acetonitrile, 0.1% formic acid) at a flow rate of 400 μL/min. Mobile phase A was kept constant for 1 min at 1%, linearly increased from 1% to 45% for 10 min, kept constant for 2 min, returned to the initial condition over 1 min, and kept constant for 4 min before the next injection. The full-HRMS mode was applied for metabolite detection (mass-to-charge ratio [m/z] range 200–1,200) at a mass resolution of 25,000 full-widths at half maximum (centroid mode). The ionization settings were as follows: capillary voltage, +3 kV (ESI+) or −2 kV (ESI−); cone voltage, 30 V; desolvation gas (N_2) flow rate, 900 L/h; and desolvation gas/source temperatures, 450°C/120°C. Leucine enkephalin solution at 2 μg/mL (50% acetonitrile) was infused at a constant flow rate of 10 μL/min in the lock spray channel, allowing for correction of the measured m/z throughout the batch. Data acquisition was achieved using MassLynx software version 4.1. For each dataset (HILIC-NEG, HILIC-POS, RP-NEG, and RP-POS), data were then converted into open source format (*.mzML) with a home-made R script (to remove lock mass) and msConvert software (version 3.0.21101-e1b2cc25b). Files were then uploaded into Galaxy workflow4metabolomics for peakpicking[56] and alignment. The batch effect was assessed and corrected using “QC samplesbatch” correction with loess method. Several filtration steps were performed, in particular based on %RSD in QC samples. A quality metrics tool was used to assess overall data quality by visualizing QC samples across each batch and through principal-component analysis. The intensity tables for each dataset (HILIC-NEG, HILIC-POS, RP-NEG, and RP-POS) were log-transformed, centered, and scaled in order to combine them for a multiblock analysis. Descriptive modeling of the global dataset was performed using PLS-DA with the mixOmics R package.[57] This allowed the representation of the samples and variables (ions) and to visualize their separation or clustering in a reduced-dimensional space. Metabolites were identified and characterized by consulting the Human Metabolome Database as well as the use of standard compounds (https://hmdb.ca/).

Quantification of amino acids by mass spectrometry

Concentrations of amino acids were determined by liquid chromatography-tandem mass spectrometry (LC-MS/MS) on a Xevo Triple-Quadrupole mass spectrometer with an ESI interface equipped with an Acquity H-Class UPLC device (Waters Corporation). All solvents used here were LC-MS grade and purchased from Biosolve. Standard compounds were obtained from Sigma-Aldrich (Saint-Quentin Fallavier, France). As mentioned previously, supernatants were obtained from DCs cultured alone in complete media for 7 days, and complete medium was used as control. Individual stock solutions (10 mmol/L) of both labeled and unlabeled amino acids were prepared in 0.1 M HCl. A pool of unlabeled standard solutions was prepared and serially diluted in water to obtain seven standard solutions ranging from 0.1 to 50 μmol/L. A pool solution of labeled amino acids (50 μmol/L) was prepared in water and used as internal standards. The standard solutions and supernatant samples (20 μL) were then extracted with 100 μL of methanol and 25 μL of the internal standard solution. The samples were mixed and centrifuged at 10,000 × g and 10°C for 15 min to remove the precipitated proteins. The supernatants were collected and dried under a gentle stream of nitrogen (45°C). A derivatization step was performed by dissolving the dried extract in 100 μL of a freshly prepared butanol solution containing 5% acetyl chloride and kept at 60°C for 30 min. The solvent was then removed under a gentle stream of nitrogen (60°C). The dried samples were dissolved in 100 μL of water containing 0.1% formic acid and 50 μmol/L TCEP and injected into the LC-MS/MS system. Samples (10 μL) were injected onto an Acquity BEH-C18 column (1.7 μm; 2.1 × 100 mm, Waters Corporation) held at 60°C, and compounds were separated with a linear gradient of mobile phase B (0.1% formic acid in methanol) in mobile phase A (0.1% formic acid in water) at a flow rate of 400 μL/min. Mobile phase B was kept constant at 1% for 0.5 min, linearly increased from 1% to 95% for 4.5 min, kept constant for 1 min, returned to the initial condition over 0.5 min, and kept constant for 1.5 min before the next injection. Target compounds were then detected by the mass spectrometer with the electrospray interface operating in the positive ion mode (capillary voltage, 3 kV; desolvation gas (N2) flow, 650 L/h; desolvation gas temperature, 350°C; source temperature, 120°C). The multiple reaction monitoring mode was applied for MS/MS detection as detailed in Table S1. Chromatographic peak area ratios between unlabeled and labeled compounds constituted the detector responses. Standard solutions were used to plot the calibration curves for quantification (linear regression, 1/x weighting, origin excluded). Data acquisition and processing were achieved using MassLynx and TargetLynx software, respectively (version 4.1, Waters Corporation).

Quantification of sphingolipids by mass spectrometry

Concentrations of sphingolipids were determined by LC-MS/MS on a Xevo Triple-Quadrupole mass spectrometer with an ESI interface equipped with an Acquity H-Class UPLC device (Waters Corporation). Standard compounds were obtained from Sigma-Aldrich. As mentioned previously, supernatants were obtained from DCs cultured alone in complete media for 7 days, and complete medium was used as control. Individual stock solutions (2.5 mmol/L) of both endogenous (C16:0, C18:0, C20:0, C22:0, C24:0, C22:1, and C24:1 ceramides [Cer]; C16:0, C24:0, and C24:1 Hexosyl-Cer; C16:0, C24:0,

and C24:1 Lactosyl-Cer; C16:0, C24:0, and C24:1 dehydro-Cer; and C14:0, C16:0, C18:0, C20:0, C22:0, C24:0, C16:1, C18:1, C20:1, C22:1, and C24:1 sphingomyelins [SMs]) and exogenous (C17:0 Cer and C17:0 SM) were prepared in isopropanol. A pool of endogenous standard solutions was prepared and serially diluted in isopropanol to obtain seven standard solutions ranging 5–5,000 and 100–100,000 nmol/L for Cer and SMs, respectively. A pool solution of exogenous compounds (5 μmol/L) was prepared in isopropanol and used as internal standards. The standard solutions and supernatant samples (50 μL) were then extracted with 500 μL of a methanol/chloroform mixture (2:1; v:v) and 50 μL of the internal standard solution. The samples were mixed and centrifuged at 10,000 × g and 10°C for 10 min. The supernatants were collected and dried under a gentle stream of nitrogen (45°C). The dried samples were dissolved in 100 μL of methanol and injected into the LC-MS/MS system. Samples (10 μL) were injected onto an Acquity BEH-C18 column (1.7 μm; 2.1 × 50 mm, Waters Corporation) held at 60°C, and compounds were separated with a linear gradient of mobile phase B (50% acetonitrile, 50% isopropanol, 0.1% formic acid, and 10 mmol/L ammonium formate) in mobile phase A (5% acetonitrile, 0.1% formic acid, and 10 mmol/L ammonium formate in water) at a flow rate of 400 μL/min. Mobile phase B was linearly increased from 40% to 99% for 4 min, kept constant for 1.5 min, returned to the initial condition over 0.5 min, and kept constant for 2 min before the next injection. Target compounds were then detected by the mass spectrometer with the electrospray interface operating in the positive ion mode (capillary voltage, 3 kV; desolvation gas (N_2) flow, 650 L/h; desolvation gas temperature, 400°C; source temperature, 150°C). The multiple reaction monitoring mode was applied for MS/MS detection as detailed in Table S2. Chromatographic peak area ratios between endogenous and exogenous compounds constituted the detector responses. Standard solutions were used to plot the calibration curves for quantification (linear regression, 1/x weighting, origin excluded). Data acquisition and processing were achieved using MassLynx and TargetLynx software, respectively (version 4.1, Waters Corporation).

Quantification of acylcarnitines by mass spectrometry

Concentrations of acylcarnitines were determined by LC-MS/MS on an Absolute Triple-Quadrupole mass spectrometer with an ESI interface equipped with an Acquity PREMIER UPLC device (Waters Corporation). All solvents used here were LC-MS grade and purchased from Biosolve. Standard compounds were obtained from Sigma-Aldrich (Saint-Quentin Fallavier, France). As mentioned previously, supernatants were obtained from DCs cultured alone in complete media for 7 days, and complete medium was used as control. Individual stock solutions (1 mmol/L) of both labeled and unlabeled acylcarnitines were prepared in methanol. A pool of unlabeled standard solutions was prepared and serially diluted in methanol to obtain seven standard solutions ranging from 20 to 5,000 nmol/L. A pool solution of labeled acylcarnitines (5 μmol/L) was prepared in methanol and used as internal standards. The standard solutions and supernatant samples (20 μL) were then extracted with 100 μL of methanol and 25 μL of the internal standard solution. The samples were mixed and centrifuged at 10,000 × g and 10°C for 15 min to remove the precipitated proteins. The supernatants were collected and dried under a gentle stream of nitrogen (45°C). A derivatization step was performed by dissolving the dried extract in 100 μL of a freshly prepared butanol solution containing 5% acetyl chloride and kept at 60°C for 30 min. The solvent was then removed under a gentle stream of nitrogen (60°C). The dried samples were dissolved in 100 μL of 25% acetonitrile containing 0.1% formic acid and injected into the LC-MS/MS system. Samples (10 μL) were injected onto an Acquity BEH-C18 column (1.7 μm; 2.1 × 50 mm, Waters Corporation) held at 60°C, and compounds were separated with a linear gradient of mobile phase B (0.1% formic acid in acetonitrile) in mobile phase A (5% acetonitrile and 0.1% formic acid in water) at a flow rate of 600 μL/min. Mobile phase B was kept constant at 10% for 0.5 min, linearly increased from 10% to 99% for 4.5 min, kept constant for 1 min, returned to the initial condition over 0.5 min, and kept constant for 1 min before the next injection. Target compounds were then detected by the mass spectrometer with the electrospray interface operating in the positive ion mode (capillary voltage, 3 kV; desolvation gas (N_2) flow, 800 L/h; desolvation gas temperature, 400°C; source temperature, 150°C). The multiple reaction monitoring mode was applied for MS/MS detection as detailed in Table S3. Chromatographic peak area ratios between unlabeled and labeled compounds constituted the detector responses. Standard solutions were used to plot the calibration curves for quantification (linear regression, 1/x weighting, origin excluded). Data acquisition and processing were achieved using MassLynx and TargetLynx software, respectively (version 4.1, Waters Corporation).

Quantification and statistical analysis

Statistical analyses for DC phenotype and functional assays were performed using GraphPad Prism. Results were expressed as the mean ± SEM, and data were assumed to follow a normal distribution. As detailed in figure legends, group comparisons were made using one-way ANOVA (paired or unpaired), Mann-Whitney *t* test, or Wilcoxon matched pairs *t* test. $*p < 0.05$, $**p < 0.01$, $***p < 0.001$. "*n*" corresponds to the number of individual donors analyzed.

DATA AND CODE AVAILABILITY

Raw data will become available upon request.

ACKNOWLEDGMENTS

We thank the institute du Thorax (Nantes university) and MELISA core facility (Oniris, Nantes), as well as SM[2] Platform (Rennes University) and M-shark platform (Nantes University) for their work. The research was supported by the EU program H2020-MSCA-ITN-2019 860003, Fondation ARC, SFNDT, FRM, and the LabEx IGO program (no. ANR-11-LABX-0016) funded by the «Investment into the Future» French Government program, managed by the National Research Agency (ANR).

AUTHOR CONTRIBUTIONS

M.S., G.A., S.G., and A.M. conceived the project and designed the experiments. M.S., G.A., M.R., M.C., C.C., and D.L. performed the experiments. M.S., G.A., M.R., M.C., A.R., T.D., and A.-L.R. analyzed the data. E.C., D.R., and F.S.d.S. contributed to the data interpretation. M.S., G.A., S.G., and A.M. wrote the manuscript.

DECLARATION OF INTERESTS

The authors declare no competing interests.

SUPPLEMENTAL INFORMATION

Supplemental information can be found online at https://doi.org/10.1016/j.omtm.2025.101605.

REFERENCES

1. Perico, N., Casiraghi, F., Introna, M., Gotti, E., Todeschini, M., Cavinato, R.A., Capelli, C., Rambaldi, A., Cassis, P., Rizzo, P., et al. (2011). Autologous mesenchymal stromal cells and kidney transplantation: a pilot study of safety and clinical feasibility. Clin. J. Am. Soc. Nephrol. *6*, 412–422. https://doi.org/10.2215/CJN.04950610.

2. Perico, N., Casiraghi, F., Gotti, E., Introna, M., Todeschini, M., Cavinato, R.A., Capelli, C., Rambaldi, A., Cassis, P., Rizzo, P., et al. (2013). Mesenchymal stromal cells and kidney transplantation: pretransplant infusion protects from graft dysfunction while fostering immunoregulation. Transpl. Int. *26*, 867–878. https://doi.org/10.1111/tri.12132.

3. Tan, J., Wu, W., Xu, X., Liao, L., Zheng, F., Messinger, S., Sun, X., Chen, J., Yang, S., Cai, J., et al. (2012). Induction therapy with autologous mesenchymal stem cells in living-related kidney transplants: a randomized controlled trial. JAMA *307*, 1169–1177. https://doi.org/10.1001/jama.2012.316.

4. Reinders, M.E.J., de Fijter, J.W., Roelofs, H., Bajema, I.M., de Vries, D.K., Schaapherder, A.F., Claas, F.H.J., van Miert, P.P.M.C., Roelen, D.L., van Kooten, C., et al. (2013). Autologous bone marrow-derived mesenchymal stromal cells for the treatment of allograft rejection after renal transplantation: results of a phase I study. Stem Cells Transl. Med. *2*, 107–111. https://doi.org/10.5966/sctm.2012-0114.

5. Peng, Y., Ke, M., Xu, L., Liu, L., Chen, X., Xia, W., Li, X., Chen, Z., Ma, J., Liao, D., et al. (2013). Donor-derived mesenchymal stem cells combined with low-dose tacrolimus prevent acute rejection after renal transplantation: a clinical pilot study. Transplantation *95*, 161–168. https://doi.org/10.1097/TP.0b013e3182754c53.

6. Mudrabettu, C., Kumar, V., Rakha, A., Yadav, A.K., Ramachandran, R., Kanwar, D.B., Nada, R., Minz, M., Sakhuja, V., Marwaha, N., and Jha, V. (2015). Safety and efficacy of autologous mesenchymal stromal cells transplantation in patients undergoing living donor kidney transplantation: a pilot study. Nephrol. Carlton Vic *20*, 25–33. https://doi.org/10.1111/nep.12338.

7. Pan, G.-H., Chen, Z., Xu, L., Zhu, J.-H., Xiang, P., Ma, J.-J., Peng, Y.-W., Li, G.-H., Chen, X.-Y., Fang, J.-L., et al. (2016). Low-dose tacrolimus combined with donor-derived mesenchymal stem cells after renal transplantation: a prospective, non-randomized study. Oncotarget *7*, 12089–12101. https://doi.org/10.18632/oncotarget.7725.

8. Perico, N., Casiraghi, F., Todeschini, M., Cortinovis, M., Gotti, E., Portalupi, V., Mister, M., Gaspari, F., Villa, A., Fiori, S., et al. (2018). Long-Term Clinical and Immunological Profile of Kidney Transplant Patients Given Mesenchymal Stromal Cell Immunotherapy. Front. Immunol. *9*, 1359. https://doi.org/10.3389/fimmu.2018.01359.

9. Bluestone, J.A., Buckner, J.H., Fitch, M., Gitelman, S.E., Gupta, S., Hellerstein, M.K., Herold, K.C., Lares, A., Lee, M.R., Li, K., et al. (2015). Type 1 diabetes immunotherapy using polyclonal regulatory T cells. Sci. Transl. Med. *7*, 315ra189. https://doi.org/10.1126/scitranslmed.aad4134.

10. Di Ianni, M., Falzetti, F., Carotti, A., Terenzi, A., Castellino, F., Bonifacio, E., Del Papa, B., Zei, T., Ostini, R.I., Cecchini, D., et al. (2011). Tregs prevent GVHD and promote immune reconstitution in HLA-haploidentical transplantation. Blood *117*, 3921–3928. https://doi.org/10.1182/blood-2010-10-311894.

11. Marek-Trzonkowska, N., Myśliwiec, M., Dobyszuk, A., Grabowska, M., Derkowska, I., Juścińska, J., Owczuk, R., Szadkowska, A., Witkowski, P., Młynarski, W., et al. (2014). Therapy of type 1 diabetes with CD4(+)CD25(high)CD127-regulatory T cells prolongs survival of pancreatic islets - results of one year follow-up. Clin. Immunol. *153*, 23–30. https://doi.org/10.1016/j.clim.2014.03.016.

12. Todo, S., Yamashita, K., Goto, R., Zaitsu, M., Nagatsu, A., Oura, T., Watanabe, M., Aoyagi, T., Suzuki, T., Shimamura, T., et al. (2016). A pilot study of operational tolerance with a regulatory T-cell-based cell therapy in living donor liver transplantation. Hepatology *64*, 632–643. https://doi.org/10.1002/hep.28459.

13. Morath, C., Schaier, M., Ibrahim, E., Wang, L., Kleist, C., Opelz, G., Süsal, C., Ponath, G., Aly, M., Alvarez, C.M., et al. (2023). Induction of Long-Lasting Regulatory B Lymphocytes by Modified Immune Cells in Kidney Transplant Recipients. J. Am. Soc. Nephrol. *34*, 160–174. https://doi.org/10.1681/ASN.2022020210.

14. Morath, C., Schmitt, A., Kleist, C., Daniel, V., Opelz, G., Süsal, C., Ibrahim, E., Kälble, F., Speer, C., Nusshag, C., et al. (2020). Phase I trial of donor-derived modified immune cell infusion in kidney transplantation. J. Clin. Investig. *130*, 2364–2376. https://doi.org/10.1172/JCI133595.

15. Sawitzki, B., Harden, P.N., Reinke, P., Moreau, A., Hutchinson, J.A., Game, D.S., Tang, Q., Guinan, E.C., Battaglia, M., Burlingham, W.J., et al. (2020). Regulatory cell therapy in kidney transplantation (The ONE Study): a harmonised design and analysis of seven non-randomised, single-arm, phase 1/2A trials. Lancet *395*, 1627–1639. https://doi.org/10.1016/S0140-6736(20)30167-7.

16. Giannoukakis, N., Phillips, B., Finegold, D., Harnaha, J., and Trucco, M. (2011). Phase I (Safety) Study of Autologous Tolerogenic Dendritic Cells in Type 1 Diabetic Patients. Diabetes Care *34*, 2026–2032. https://doi.org/10.2337/dc11-0472.

17. Zubizarreta, I., Flórez-Grau, G., Vila, G., Cabezón, R., España, C., Andorra, M., Saiz, A., Llufriu, S., Sepulveda, M., Sola-Valls, N., et al. (2019). Immune tolerance in multiple sclerosis and neuromyelitis optica with peptide-loaded tolerogenic dendritic cells in a phase 1b trial. Proc. Natl. Acad. Sci. USA *116*, 8463–8470. https://doi.org/10.1073/pnas.1820039116.

18. Kurochkina, Y., Tikhonova, M., Tyrinova, T., Leplina, O., Sizikov, A., Sulutian, A., Chumasova, O., Ostanin, A., and Chernykh, E. (2018). SAT0212 The safety and tolerability of intra-articular injection of tolerogenic dendritic cells in patients with rheumatoid arthritis: the preliminary results. Ann. Rheum. Dis. *77*, 966–967.

19. Benham, H., Nel, H.J., Law, S.C., Mehdi, A.M., Street, S., Ramnoruth, N., Pahau, H., Lee, B.T., Ng, J., Brunck, M.E.G., et al. (2015). Citrullinated peptide dendritic cell immunotherapy in HLA risk genotype-positive rheumatoid arthritis patients. Sci. Transl. Med. *7*, 290ra87. https://doi.org/10.1126/scitranslmed.aaa9301.

20. Bell, G.M., Anderson, A.E., Diboll, J., Reece, R., Eltherington, O., Harry, R.A., Fouweather, T., MacDonald, C., Chadwick, T., McColl, E., et al. (2017). Autologous tolerogenic dendritic cells for rheumatoid and inflammatory arthritis. Ann. Rheum. Dis. *76*, 227–234. https://doi.org/10.1136/annrheumdis-2015-208456.

21. Willekens, B., Presas-Rodríguez, S., Mansilla, M.J., Derdelinckx, J., Lee, W.-P., Nijs, G., De Laere, M., Wens, I., Cras, P., Parizel, P., et al. (2019). Tolerogenic dendritic cell-based treatment for multiple sclerosis (MS): a harmonised study protocol for two phase I clinical trials comparing intradermal and intranodal cell administration. BMJ Open *9*, e030309. https://doi.org/10.1136/bmjopen-2019-030309.

22. Jauregui-Amezaga, A., Cabezón, R., Ramírez-Morros, A., España, C., Rimola, J., Bru, C., Pinó-Donnay, S., Gallego, M., Masamunt, M.C., Ordás, I., et al. (2015). Intraperitoneal Administration of Autologous Tolerogenic Dendritic Cells for Refractory Crohn's Disease: A Phase I Study. J. Crohns Colitis *9*, 1071–1078. https://doi.org/10.1093/ecco-jcc/jjv144.

23. Bouchet-Delbos, L., Even, A., Varey, E., Saïagh, S., Bercegeay, S., Braudeau, C., Dréno, B., Blancho, G., Josien, R., Cuturi, M.-C., and Moreau, A. (2021). Preclinical assessment of Autologous Tolerogenic Dendritic Cells from End-stage renal disease patients. Transplantation *105*, 832–841. https://doi.org/10.1097/TP.0000000000003315.

24. Comi, M., Amodio, G., Passeri, L., Fortunato, M., Santoni de Sio, F.R., Andolfi, G., Kajaste-Rudnitski, A., Russo, F., Cesana, L., and Gregori, S. (2020). Generation of Powerful Human Tolerogenic Dendritic Cells by Lentiviral-Mediated IL-10 Gene Transfer. Front. Immunol. *11*, 1260. https://doi.org/10.3389/fimmu.2020.01260.

25. Morante-Palacios, O., Fondelli, F., Ballestar, E., and Martínez-Cáceres, E.M. (2021). Tolerogenic Dendritic Cells in Autoimmunity and Inflammatory Diseases. Trends Immunol. *42*, 59–75. https://doi.org/10.1016/j.it.2020.11.001.

26. Marín, E., Cuturi, M.C., and Moreau, A. (2018). Tolerogenic Dendritic Cells in Solid Organ Transplantation: Where Do We Stand? Front. Immunol. *9*, 274. https://doi.org/10.3389/fimmu.2018.00274.

27. Marin, E., Bouchet-Delbos, L., Renoult, O., Louvet, C., Nerriere-Daguin, V., Managh, A.J., Even, A., Giraud, M., Vu Manh, T.P., Aguesse, A., et al. (2019). Human Tolerogenic Dendritic Cells Regulate Immune Responses through Lactate Synthesis. Cell Metab. *30*, 1075–1090.e8. https://doi.org/10.1016/j.cmet.2019.11.011.

28. Svajger, U., Obermajer, N., and Jeras, M. (2010). Novel findings in drug-induced dendritic cell tolerogenicity. Int. Rev. Immunol. *29*, 574–607. https://doi.org/10.3109/08830185.2010.522280.

29. Gordon, J.R., Ma, Y., Churchman, L., Gordon, S.A., and Dawicki, W. (2014). Regulatory dendritic cells for immunotherapy in immunologic diseases. Front. Immunol. *5*, 7. https://doi.org/10.3389/fimmu.2014.00007.

30. Piemonti, L., Monti, P., Allavena, P., Sironi, M., Soldini, L., Leone, B.E., Socci, C., and Di Carlo, V. (1999). Glucocorticoids affect human dendritic cell differentiation and maturation. J. Immunol. *162*, 6473–6481.

31. Lagaraine, C., Hoarau, C., Chabot, V., Velge-Roussel, F., and Lebranchu, Y. (2005). Mycophenolic acid-treated human dendritic cells have a mature migratory phenotype and inhibit allogeneic responses via direct and indirect pathways. Int. Immunol. *17*, 351–363. https://doi.org/10.1093/intimm/dxh215.

32. Li, X.-L., Liu, Y., Cao, L.-L., Li, H., Yue, L.-T., Wang, S., Zhang, M., Li, X.-H., Dou, Y.-C., and Duan, R.-S. (2013). Atorvastatin-modified dendritic cells in vitro ameliorate experimental autoimmune myasthenia gravis by up-regulated Treg cells and shifted Th1/Th17 to Th2 cytokines. Mol. Cell. Neurosci. *56*, 85–95. https://doi.org/10.1016/j.mcn.2013.03.005.

33. Bhatt, S., Qin, J., Bennett, C., Qian, S., Fung, J.J., Hamilton, T.A., and Lu, L. (2014). All-trans retinoic acid induces arginase-1 and inducible nitric oxide synthase-producing dendritic cells with T cell inhibitory function. J. Immunol. *192*, 5098–5108. https://doi.org/10.4049/jimmunol.1303073.

34. Kim, S.H., Jung, H.H., and Lee, C.K. (2018). Generation, Characteristics and Clinical Trials of Ex Vivo Generated Tolerogenic Dendritic Cells. Yonsei Med. J. *59*, 807–815. https://doi.org/10.3349/ymj.2018.59.7.807.

35. Torres-Aguilar, H., Aguilar-Ruiz, S.R., González-Pérez, G., Munguía, R., Bajaña, S., Meraz-Ríos, M.A., and Sánchez-Torres, C. (2010). Tolerogenic dendritic cells generated with different immunosuppressive cytokines induce antigen-specific anergy and regulatory properties in memory CD4+ T cells. J. Immunol. *184*, 1765–1775. https://doi.org/10.4049/jimmunol.0902133.

36. Nayyar, A., Dawicki, W., Huang, H., Lu, M., Zhang, X., and Gordon, J.R. (2012). Induction of prolonged asthma tolerance by IL-10-differentiated dendritic cells: differential impact on airway hyperresponsiveness and the Th2 immunoinflammatory response. J. Immunol. *189*, 72–79. https://doi.org/10.4049/jimmunol.1103286.

37. Moreau, A., Varey, E., Bouchet-Delbos, L., and Cuturi, M.-C. (2012). Cell therapy using tolerogenic dendritic cells in transplantation. Transplant. Res. *1*, 13. https://doi.org/10.1186/2047-1440-1-13.

38. Gregori, S., Tomasoni, D., Pacciani, V., Scirpoli, M., Battaglia, M., Magnani, C.F., Hauben, E., and Roncarolo, M.-G. (2010). Differentiation of type 1 T regulatory cells (Tr1) by tolerogenic DC-10 requires the IL-10-dependent ILT4/HLA-G pathway. Blood *116*, 935–944. https://doi.org/10.1182/blood-2009-07-234872.

39. Moreau, A., Hill, M., Thébault, P., Deschamps, J.Y., Chiffoleau, E., Chauveau, C., Moullier, P., Anegon, I., Alliot-Licht, B., and Cuturi, M.C. (2009). Tolerogenic dendritic cells actively inhibit T cells through heme oxygenase-1 in rodents and in nonhuman primates. FASEB J. Off. Publ. Fed. Am. Soc. Exp. Biol. *23*, 3070–3077. https://doi.org/10.1096/fj.08-128173.

40. Moreau, A., Vandamme, C., Segovia, M., Devaux, M., Guilbaud, M., Tilly, G., Jaulin, N., Le Duff, J., Cherel, Y., Deschamps, J.-Y., et al. (2014). Generation and in vivo evaluation of IL10-treated dendritic cells in a nonhuman primate model of AAV-based gene transfer. Mol. Ther. Methods Clin. Dev. *1*, 14028. https://doi.org/10.1038/mtm.2014.28.

41. Moreau, A., Kervella, D., Bouchet-Delbos, L., Braudeau, C., Saïagh, S., Guérif, P., Limou, S., Moreau, A., Bercegeay, S., Streitz, M., et al. (2023). A Phase I/IIa study of autologous tolerogenic dendritic cells immunotherapy in kidney transplant recipients. Kidney Int. *103*, 627–637. https://doi.org/10.1016/j.kint.2022.08.037.

42. Lutz, M.B., Suri, R.M., Niimi, M., Ogilvie, A.L., Kukutsch, N.A., Rössner, S., Schuler, G., and Austyn, J.M. (2000). Immature dendritic cells generated with low doses of GM-CSF in the absence of IL-4 are maturation resistant and prolong allograft survival in vivo. Eur. J. Immunol. *30*, 1813–1822. https://doi.org/10.1002/1521-4141(200007)30:7<1813::AID-IMMU1813>3.0.CO;2-8.

43. Levings, M.K., Gregori, S., Tresoldi, E., Cazzaniga, S., Bonini, C., and Roncarolo, M.G. (2005). Differentiation of Tr1 cells by immature dendritic cells requires IL-10 but not CD25+CD4+ Tr cells. Blood *105*, 1162–1169. https://doi.org/10.1182/blood-2004-03-1211.

44. Schmidt, M., Lügering, N., Pauels, H.G., Schulze-Osthoff, K., Domschke, W., and Kucharzik, T. (2000). IL-10 induces apoptosis in human monocytes involving the CD95 receptor/ligand pathway. Eur. J. Immunol. *30*, 1769–1777. https://doi.org/10.1002/1521-4141(200006)30:6<1769::AID-IMMU1769>3.0.CO;2-9.

45. Chang, W.L.W., Baumgarth, N., Eberhardt, M.K., Lee, C.Y.D., Baron, C.A., Gregg, J.P., and Barry, P.A. (2007). Exposure of myeloid dendritic cells to exogenous or endogenous IL-10 during maturation determines their longevity. J. Immunol. *178*, 7794–7804. https://doi.org/10.4049/jimmunol.178.12.7794.

46. Hashimoto, S.I., Komuro, I., Yamada, M., and Akagawa, K.S. (2001). IL-10 inhibits granulocyte-macrophage colony-stimulating factor-dependent human monocyte survival at the early stage of the culture and inhibits the generation of macrophages. J. Immunol. *167*, 3619–3625. https://doi.org/10.4049/jimmunol.167.7.3619.

47. Comi, M., Amodio, G., and Gregori, S. (2018). Interleukin-10-Producing DC-10 Is a Unique Tool to Promote Tolerance Via Antigen-Specific T Regulatory Type 1 Cells. Front. Immunol. *9*, 682. https://doi.org/10.3389/fimmu.2018.00682.

48. Roncarolo, M.G., Gregori, S., Bacchetta, R., Battaglia, M., and Gagliani, N. (2018). The Biology of T Regulatory Type 1 Cells and Their Therapeutic Application in Immune-Mediated Diseases. Immunity *49*, 1004–1019. https://doi.org/10.1016/j.immuni.2018.12.001.

49. Dawson, H., Collins, G., Pyle, R., Deep-Dixit, V., and Taub, D.D. (2004). The immunoregulatory effects of homocysteine and its intermediates on T-lymphocyte function. Mech. Ageing Dev. *125*, 107–110. https://doi.org/10.1016/j.mad.2003.11.013.

50. Dai, J., and Wang, X. (2007). Immunoregulatory effects of homocysteine on cardiovascular diseases. Sheng Li Xue Bao *59*, 585–592.

51. Aguayo-Cerón, K.A., Sánchez-Muñoz, F., Gutierrez-Rojas, R.A., Acevedo-Villavicencio, L.N., Flores-Zarate, A.V., Huang, F., Giacoman-Martinez, A., Villafaña, S., and Romero-Nava, R. (2023). Glycine: The Smallest Anti-Inflammatory Micronutrient. Int. J. Mol. Sci. *24*, 11236. https://doi.org/10.3390/ijms241411236.

52. Zhang, D., Tang, Z., Huang, H., Zhou, G., Cui, C., Weng, Y., Liu, W., Kim, S., Lee, S., Perez-Neut, M., et al. (2019). Metabolic regulation of gene expression by histone lactylation. Nature *574*, 575–580. https://doi.org/10.1038/s41586-019-1678-1.

53. Ganeshan, K., and Chawla, A. (2014). Metabolic regulation of immune responses. Annu. Rev. Immunol. *32*, 609–634. https://doi.org/10.1146/annurev-immunol-032713-120236.

54. Morali, K., Giacomello, G., Vuono, M., and Gregori, S. (2025). Leveraging current insights on IL-10-producing dendritic cells for developing effective immunotherapeutic approaches. FEBS Lett. *599*, 2025–2047. https://doi.org/10.1002/1873-3468.15017.

55. Mansilla, M.J., González-Larreategui, I., Figa-Martín, N., Barallat, J., Fondelli, F., Sellés-Rius, A., Quirant-Sánchez, B., Teniente-Serra, A., and Martínez-Cáceres, E. (2021). Transfection of Vitamin D3-Induced Tolerogenic Dendritic Cells for the Silencing of Potential Tolerogenic Genes. Identification of CSF1R-CSF1 Signaling as a Glycolytic Regulator. Int. J. Mol. Sci. *22*, 7363. https://doi.org/10.3390/ijms22147363.

56. Giacomoni, F., Le Corguillé, G., Monsoor, M., Landi, M., Pericard, P., Pétéra, M., Duperier, C., Tremblay-Franco, M., Martin, J.-F., Jacob, D., et al. (2015). Workflow4Metabolomics: a collaborative research infrastructure for computational metabolomics. Bioinformatics *31*, 1493–1495. https://doi.org/10.1093/bioinformatics/btu813.

57. Rohart, F., Gautier, B., Singh, A., and Lê Cao, K.-A. (2017). mixOmics: An R package for ’omics feature selection and multiple data integration. PLoS Comput. Biol. *13*, e1005752. https://doi.org/10.1371/journal.pcbi.1005752.

OMTM, Volume *33*

Supplemental information

Protocol of generation of tolerogenic dendritic cells affects their transcriptional and metabolic profiles leading to specific tolerogenic functions

Maaike Suuring, Giada Amodio, Mathieu Rouel, Axel Raux, Thomas Delhaye, Anne-Lise Royer, Chloé Cloteau, Denisia Lavercan, David Rondeau, Elise Chiffoleau, Francesca Santoni de Sio, Mikaël Croyal, Silvia Gregori, and Aurélie Moreau

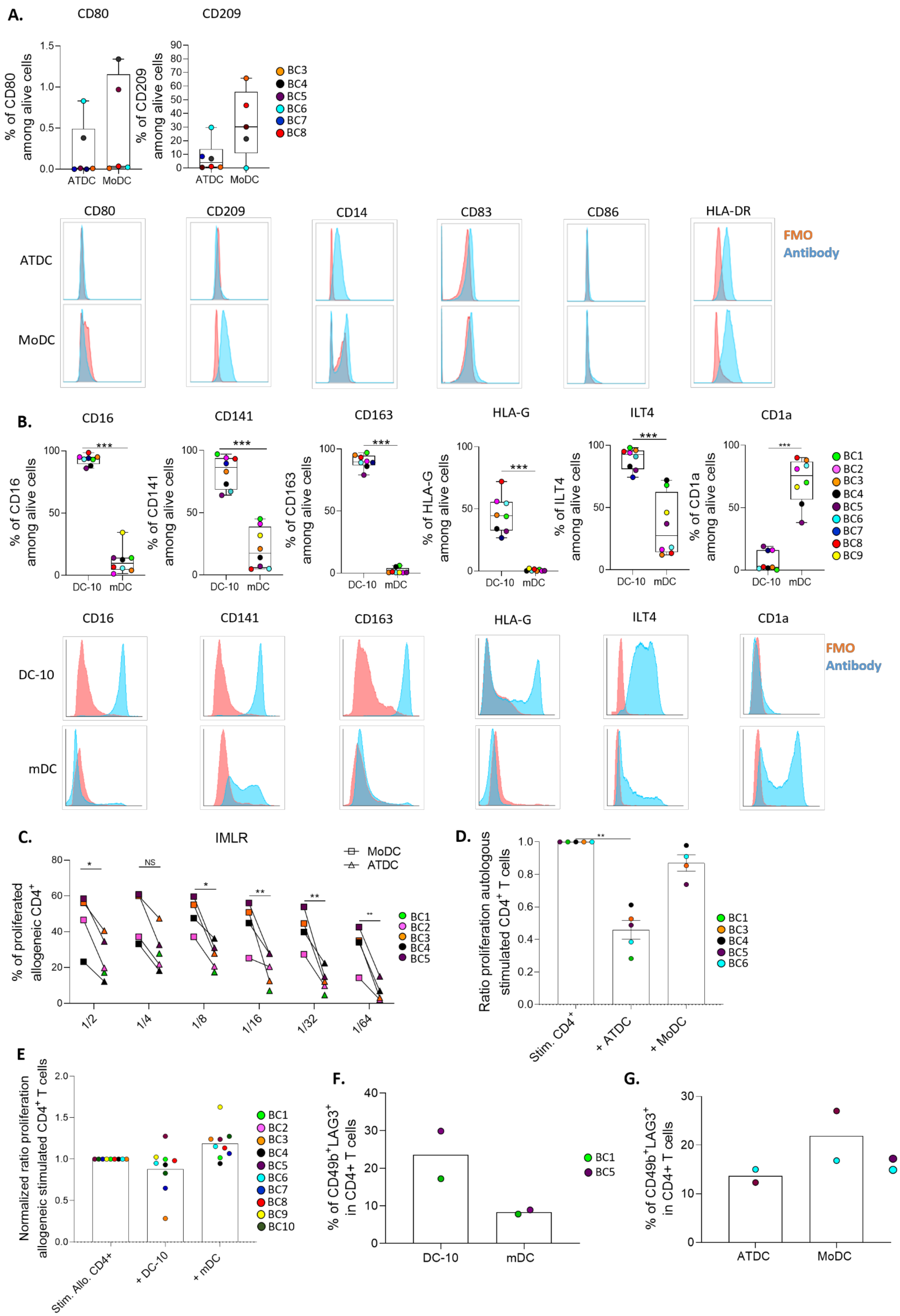

A.
CD80
CD209
% of CD80 among alive cells
% of CD209 among alive cells
ATDC
MoDC
BC3
BC4
BC5
BC6
BC7
BC8
CD14
CD83
CD86
HLA-DR
FMO
Antibody
B.
CD16
CD141
CD163
HLA-G
ILT4
CD1a
% of CD16 among alive cells
% of CD141 among alive cells
% of CD163 among alive cells
% of HLA-G among alive cells
% of ILT4 among alive cells
% of CD1a among alive cells
DC-10
mDC
BC1
BC2
BC9
C.
IMLR
% of proliferated allogeneic CD4+
MoDC
ATDC
NS
1/2
1/4
1/8
1/16
1/32
1/64
D.
Ratio proliferation autologous stimulated CD4+ T cells
Stim. CD4+
+ ATDC
+ MoDC
E
Normalized ratio proliferation allogeneic stimulated CD4+ T cells
Stim. Allo. CD4+
+ DC-10
+ mDC
BC10
F.
% of CD49b+LAG3+ in CD4+ T cells
G.

Figure S1 – ATDC and DC-10 display distinct phenotype and tolerogenic functions *(related to Figure 2)*

(A) Frequency (%) of cells expressing the indicated markers among alive cells in ATDC and MoDC ($n \geq 5$ donors). Histograms of the markers expressed by ATDC and MoDC (blue) and their respective FMO controls (orange). **(B)** Bar charts of the frequency (%) of cells expressing the indicated markers among alive cells in DC-10 and mDC ($n = 8$ donors). **(C)** DCs were cultured with allogeneic CPD labeled $CD4^+$ T cells at the indicated T:DC ratios. At day 5, T cell proliferation was assessed by CPD dilution ($n \geq 6$ donors). **(D and E)** CPD labeled $CD4^+$ T cells were cultured with allogeneic mature DCs at 1:10 T:DC ratio in the presence or absence of ATDC and MoDC (panel D) or DC-10 and mDC (panel E). At day 5, T cell proliferation was assessed by CPD dilution ($n = 5$ donors). **(F and G)** $CD4^+$ T cells were cultured with allogeneic DCs, either DC-10 or mDC (panel F), or ATDC or MoDC (panel G), at 1:10 ratio for 10 days. At the end of the culture the frequency of Tr1 cells was analyzed by the co-expression of LAG-3 and CD49b by flow cytometry ($n = 2$ donors). p values were calculated by Mann-Whitney T-test (A - B), two-way ANOVA (C) and Kruskal-Wallis test (D)*$p < 0.05$, **$p < 0.01$,***$p < 0.001$.

A.

DC-10 VS ATDC

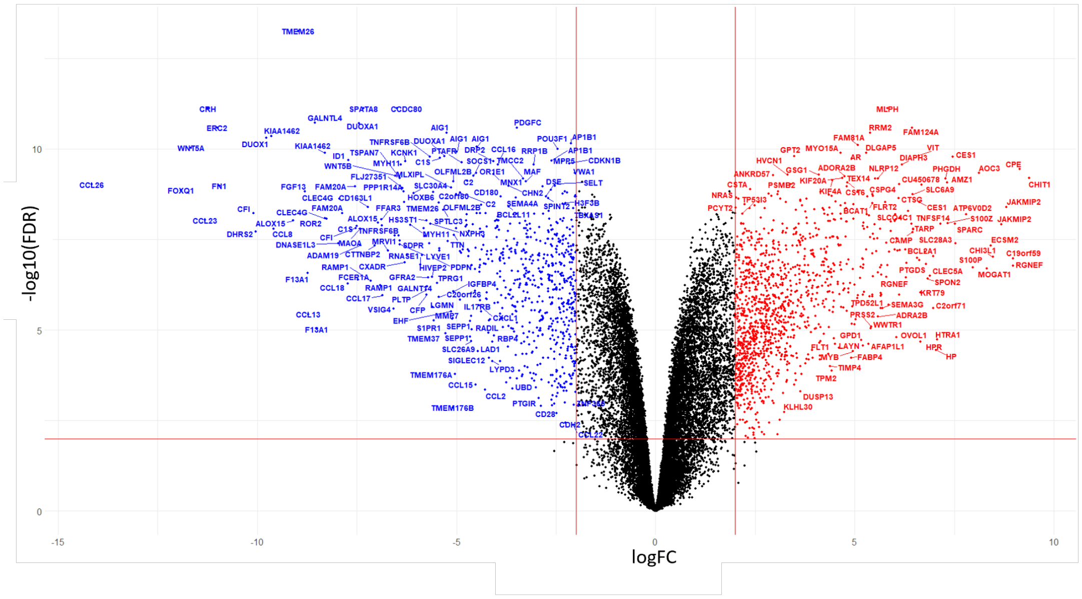

B.

MoDC VS ATDC

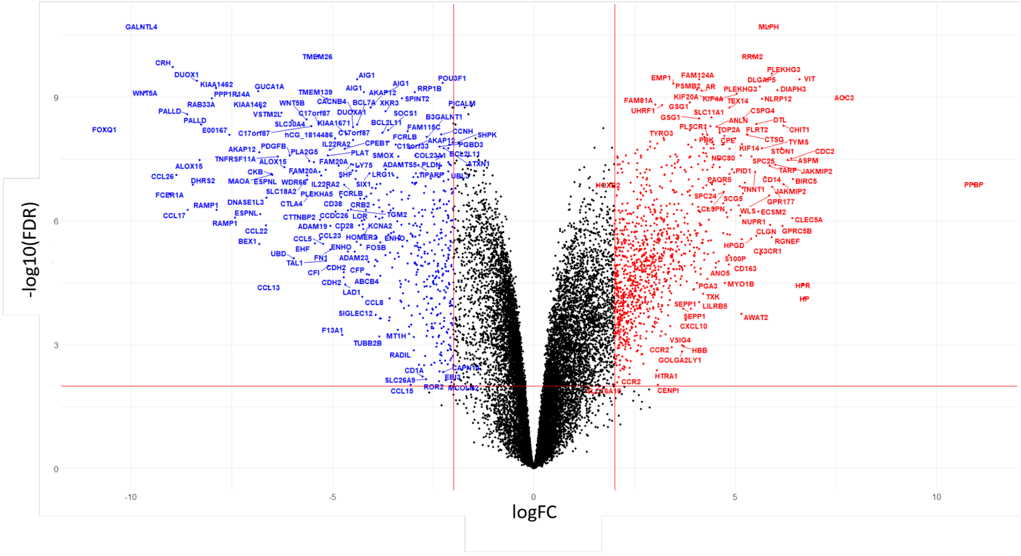

MoDC VS DC-10

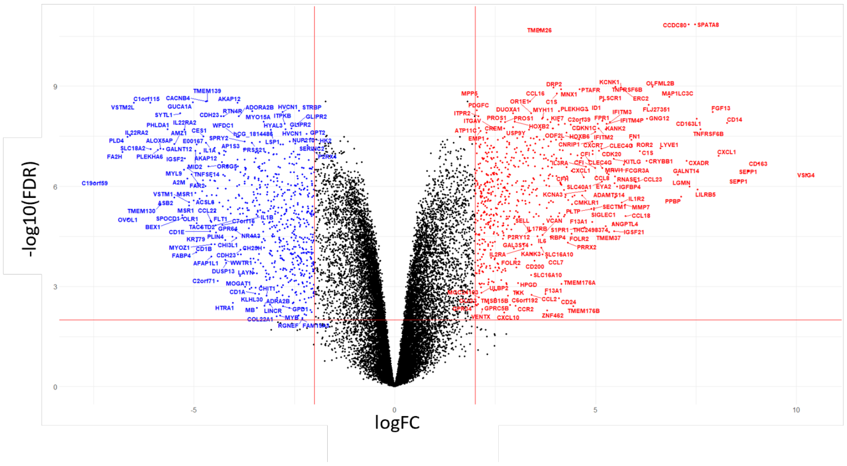

Figure S2 –Volcano plots associated to the transcriptomic analyses *(related to Figure 5)*

(A) Volcano plot illustrates the genes differentially expressed between ATDC and DC-10 ($n \geq 6$ donors). Genes upregulated in DC-10 are depicted on the left (blue). **(B)** Volcano plots illustrate the genes differentially expressed between either ATDC (up) or DC-10 (down) and MoDC ($n \geq 6$ donors). Genes upregulated in MoDC are depicted on the left (blue).

Table S1 – Multiple Reaction monitoring parameters used for the quantification of amino acids by mass spectrometry.

Amino acid	Parent ion (m/z	Fragment ion (m/z)	Cone (V)	Collision (eV)
Aspartate	246.1	144	30	20
^{15}N-aspartate (IS)	247.1	145	30	20
Glutamate	260.1	83.9	30	20
^{15}N-Glutamate (IS)	261.1	84.9	30	20
Alanine	146.1	56.9	30	15
^{13}C-Alanine (IS)	147.1	56.9	30	15
Arginine	231.1	69.9	30	20
^{13}C-Arginine (IS)	232.1	69.9	30	20
Asparagine	189.1	73.9	30	20
$^{15}N_2$-Asparagine (IS)	191.1	74.9	30	20
Cysteine	178.1	75.9	30	15
d_3-Cysteine (IS)	181.1	78.9	30	15
Glutamine	203.1	83.9	30	20
^{15}N-Glutamine (IS)	204.1	84.9	30	20
Glycine	132.1	56.9	30	15
^{13}C-Glycine (IS)	133.1	56.9	30	15
Histidine	212.2	109.9	30	20
$^{15}N_3$-Histidine (IS)	215.2	112.9	30	20
Isoleucine / Leucine	188.2	85.9	30	15
^{15}N-Leucine (IS)	189.2	86.9	30	15
Lysine	203.1	83.9	30	20
$^{13}C_6$-Lysine (IS)	209.1	88.9	30	15
Methionine	206.2	103.9	30	15
^{13}C-d_3-methionine (IS)	210.2	107.9	30	15
Phenylalanine	222.2	120.2	30	20
d_5-Phenylalanine (IS)	227.2	125.2	30	20
Proline	172.1	69.9	30	20
^{13}C-Proline (IS)	173.1	70.9	30	20

Serine	162.1	59.9	30	15
^{15}N-Serine (IS)	163.1	60.9	30	15
Threonine	176.1	73.9	30	15
^{15}N-Threonine (IS)	177.1	74.9	30	15
Tryptophane	261.2	132	30	20
d_5-Tryptophane (IS)	266.2	137	30	20
Tyrosine	238.2	136	30	20
^{15}N-Tyrosine (IS)	239.2	137	30	20
Valine	174.1	71.9	30	20
^{13}C-Valine (IS)	175.1	71.9	30	20

IS, internal standard.

Table S2 – Multiple Reaction monitoring parameters used for the quantification of sphingolipids by mass spectrometry.

**Sphingolipids**	**Parent ion (m/z**	**Fragment ion (m/z)**	**Cone (V)**	**Collision (eV)**
Cer (d18:1/16:0)	538.5	264.3	28	26
Cer (d18:1/18:0)	566.5	264.3	30	26
Cer (d18:1/20:0)	594.3	264.3	28	26
Cer (d18:1/22:0)	622.6	264.3	30	30
Cer (d18:1/24:0)	650.6	264.3	34	26
SM (d18:1/16:0)	703.6	184.1	58	26
SM (d18:1/18:0)	731.5	184.1	58	32
SM (d18:1/20:0)	759.7	184.1	46	26
SM (d18:1/22:0)	787.7	184.1	40	30
SM (d18:1/24:0)	815.7	184.1	36	30

IS, internal standard.

Table S3 – Multiple Reaction monitoring parameters used for the quantification of acylcarnitines by mass spectrometry.

**Acylcarnitines**	**Parent ion (m/z**	**Fragment ion (m/z)**	**Cone (V)**	**Collision (eV)**
Ethyl-carnitine (C2)	260.4	85.1	30	21
Propanoyl-carnitine (C3)	274.4	85.1	30	21
Butyryl-carnitine (C4)	288.4	85.1	30	21
Lauroyl-carnitine (C12)	400.5	85.1	30	24

IS, internal standard.
